# The structure of denisovite, a fibrous nanocrystalline polytypic disordered ‘very complex’ silicate, studied by a synergistic multi-disciplinary approach employing methods of electron crystallography and X-ray powder diffraction

**DOI:** 10.1107/S2052252517002585

**Published:** 2017-03-08

**Authors:** Ira V. Rozhdestvenskaya, Enrico Mugnaioli, Marco Schowalter, Martin U. Schmidt, Michael Czank, Wulf Depmeier, Andreas Rosenauer

**Affiliations:** a Department of Crystallography, Institute of Earth Science, Saint Petersburg State University, University emb. 7/9, St Petersburg 199034, Russian Federation; b Department of Physical Sciences, Earth and Environment, University of Siena, Via Laterino 8, Siena 53100, Italy; c Center for Nanotechnology Innovation@NEST, Istituto Italiano di Tecnologia, Piazza San Silvestro 12, Pisa 56127, Italy; d Institute of Solid State Physics, University of Bremen, Otto-Hahn-Allee 1, Bremen D-28359, Germany; e Institut für Anorganische und Analytische Chemie, Goethe-Universität, Max-von-Laue-Strasse 7, Frankfurt am Main D-60438, Germany; f Institute of Geosciences, Kiel University, Olshausenstrasse 40, Kiel D-24098, Germany

**Keywords:** denisovite, minerals, fibrous materials, nanocrystalline materials, electron crystallography, electron diffraction tomography, X-ray powder diffraction, modularity, disorder, polytypism, OD approach, complexity, framework-structured solids, inorganic materials, nanostructure, nanoscience

## Abstract

Denisovite is a rare mineral occurring as aggregates of fibres typically 200–500 nm diameter. It was confirmed as a new mineral in 1984, but important facts about its chemical formula, lattice parameters, symmetry and structure have remained incompletely known since then. Recently obtained results from studies using microprobe analysis, X-ray powder diffraction (XRPD), electron crystallography, modelling and Rietveld refinement will be reported. The electron crystallography methods include transmission electron microscopy (TEM), selected-area electron diffraction (SAED), high-angle annular dark-field imaging (HAADF), high-resolution transmission electron microscopy (HRTEM), precession electron diffraction (PED) and electron diffraction tomography (EDT). A structural model of denisovite was developed from HAADF images and later completed on the basis of quasi-kinematic EDT data by *ab initio* structure solution using direct methods and least-squares refinement. The model was confirmed by Rietveld refinement. The lattice parameters are *a* = 31.024 (1), *b* = 19.554 (1) and *c* = 7.1441 (5) Å, β = 95.99 (3)°, *V* = 4310.1 (5) Å^3^ and space group *P*12/*a*1. The structure consists of three topologically distinct dreier silicate chains, *viz*. two xonotlite-like dreier double chains, [Si_6_O_17_]^10−^, and a tubular loop-branched dreier triple chain, [Si_12_O_30_]^12−^. The silicate chains occur between three walls of edge-sharing (Ca,Na) octahedra. The chains of silicate tetrahedra and the octahedra walls extend parallel to the *z* axis and form a layer parallel to (100). Water molecules and K^+^ cations are located at the centre of the tubular silicate chain. The latter also occupy positions close to the centres of eight-membered rings in the silicate chains. The silicate chains are geometrically constrained by neighbouring octahedra walls and present an ambiguity with respect to their *z* position along these walls, with displacements between neighbouring layers being either Δ*z* = *c*/4 or −*c*/4. Such behaviour is typical for polytypic sequences and leads to disorder along [100]. In fact, the diffraction pattern does not show any sharp reflections with *l* odd, but continuous diffuse streaks parallel to a* instead. Only reflections with *l* even are sharp. The diffuse scattering is caused by (100) nano­lamellae separated by stacking faults and twin boundaries. The structure can be described according to the order–disorder (OD) theory as a stacking of layers parallel to (100).

## Abstract 2

Denisovite is a rare mineral occurring as fibres typically 200–500 nm in diameter, always characterized by pervasive disorder. Combining X-ray powder diffraction, high-angle annular dark-field scanning transmission electron microscopy imaging and electron diffraction tomography, it was possible to determine the denisovite structure and describe its disorder and polytypism by order–disorder theory. The denisovite structure ranks among the top 1% of the most complex mineral structures known to date.

## 1. Introduction   

For some chemists, physicists, biologists or even crystallographers, the knowledge of minerals is possibly limited to what they have learned during their early undergraduate studies, and therefore they might have developed the idea that minerals are easy objects having high symmetry, small unit cells and simple chemistry. Typical examples would then include diamond (C), halite (NaCl), sphalerite and wurtzite (ZnS) or fluorite (CaF_2_). However, most of the more than 5000 mineral species known to date are much more complicated: many have low symmetry or large unit cells, or show intricate and variable chemical compositions. One of these more complicated minerals is denisovite. In order to aid less-experienced readers in appreciating the present investigation, some significant particularities of minerals in general, and of denisovite in particular, will be summarized first.

In contrast with the majority of objects studied by ‘small-molecule’ or ‘macromolecular’ crystallography, most minerals are not composed of molecules as building blocks. Additionally, unlike typical molecules which are composed of integer numbers of atoms and thus have well defined stoichiometry, different chemical species may be randomly distributed over equivalent positions throughout the structure of many minerals. By averaging over all these positions, a typical diffraction experiment will reveal virtual species in the unit cell which represent a weighted average over the elemental species involved. Therefore, such mixed crystals, or solid solutions, often have non-integer stoichiometry. A certain prerequisite for this isomorphous replacement is that the species involved have similar sizes. A prominent example is olivine, (Mg,Fe)_2_SiO_4_, where Fe^2+^ and Mg^2+^ share the same crystallographically independent sites in the unit cell. This is indicated in the formula by grouping their chemical symbols in parentheses. One important objective of a structure determination is to quantify the ratio of these elements expressed as occupancies, *e.g.* (Mg*_x_*Fe_1−*x*_)_2_SiO_4_.

It is a notable feature of many minerals that even differently charged but similarly sized species can substitute for each other, *e.g.* Ca^2+^ and Na^+^, or OH^−^ and O^2−^, and both of these mechanisms act in denisovite. In order to maintain overall charge balance, coupled substitution mechanisms may then become necessary. A well known example is Ca^2+^ → Na^+^ coupled with Al^3+^ → Si^4+^, as in the case of plagioclase feldspars, the most common minerals of the Earth’s crust. Vacancies and oxidation/reduction of suitable species may also contribute to maintaining charge balance.

In some cases the mixing of species is ideal, or nearly so, as in olivine where 0 ≤ *x* ≤ 1. In other cases, *x* may be limited to finite ranges, often close to the end members (*x* = 0 or 1 for binary systems), or so-called miscibility gaps may occur for certain ranges of *x*. The latter are very important for understanding the properties of the above-mentioned feldspars. In many of the more complicated minerals, more than two elements may share the same site, with the occupancies of the minority elements often dwindling down to the low percent range or even as far as the level of trace amounts or impurities.

It goes without saying that different crystallographically independent positions can be occupied by the same kinds of atom. This is also true for mixed species, *e.g.* in orthorhombic olivine the (Mg,Fe) ‘species’ occupies two symmetrically independent positions with almost identical occupancies. When the components in a solid solution differ more strongly in their chemical character they may develop a preference for certain positions on the basis of more suitable site symmetry, size or neighbourhood. An example from the mineral kingdom is once more olivine. When the mineral also contains Ca^2+^, this will barely mix with Mg^2+^ and Fe^2+^ because of its significantly larger size, and it will occupy preferentially or even completely either crystallographically independent position. Elements occurring in different valence states, such as Fe^2+^ and Fe^3+^, may also coexist in the same crystal structure. A prominent example is magnetite, Fe^2+^Fe^3+^_2_O_4_. Water is often part of an extended network of hydrogen bonds, or it occupies as virtually isolated species void spaces in an otherwise extended framework structure. This is also the case for denisovite. Altogether these effects may result in sometimes rather awesome formulae, as *e.g.* in that of the mineral steenstrupine which, according to Krivovichev (2013 ▸), reads (Th_0.42_Zr_0.41_Ti_0.1_Al_0.07_)(Mn_1.49_Ca_0.51_)(Fe_1.69_Mn_0.31_)(Na_1.47_Ca_0.53_)(La_19.9_Ce_2.89_Pr_0.23_Nd_0.71_Y_0.19_)Na_12_((P_0.77_Si_0.23_)O_4_)_6_(Si_6_O_18_)_2_(PO_4_)_0.88_(OH)_2_(H_2_O)_2.19_.

From these remarks, some readers might perhaps get the impression that minerals are in fact ‘dirty’ chemicals and it would be more reasonable to deal with pure chemicals purchased from a renowned company. Indeed, this might be true for certain fields, but surely not for others. As a counter­example we mention that it is just the trace amounts of ‘impurities’ like chromium or iron and titanium, respectively, which give ruby or sapphire their specific red or blue colour and thus turn ordinary corundum into valuable highly appreciated gemstones. Perhaps even more important for our daily life is the fact that doping with ‘impurities’, including vacancies, enables materials scientists to tune the properties of many solid-state materials, thus making them useful or even indispensable utilities of our modern civilization.

The ‘mixing’ of the various species in a crystal does not necessarily happen at random, but obeys crystal-chemical rules which may allow only certain combinations. Miscibility gaps have already been mentioned. It is not uncommon to find mixed crystals, including minerals, in which, under certain conditions of temperature and pressure, or as a function of time, their components segregate into separate more or less ordered areas. Often only partial or short-range order develops, giving rise to various types of diffuse scattering. In recent years greatly improved radiation sources, instrumentation and detectors, as well as the development of sophisticated analytical methods, have become available, allowing the structural details of these often technologically important materials to be elucidated (see *e.g.* Whitfield *et al.*, 2016 ▸).

Chemical ordering may also happen on longer length scales, such that long-range ordered structures develop. Often, such ordering schemes can be regarded as periodic modulations of an underlying non-modulated basic structure. The modulations have wavelengths which are typically of the order of a few basic lattice parameters and may be commensurate or incommensurate with the latter. Apart from chemical ordering, other mechanisms are also known to trigger the formation of modulated structures, *e.g.* frustrated interaction of different structural units, or charge-density waves. In any case, a modulation results in the occurrence of extra reflections (satellites) in the diffraction pattern, in addition to the usual Bragg peaks which are due to the basic structure. For incommensurately modulated structures it is not possible to index all reflections, in particular the satellites, with three integer indices as required for ordinary crystals with three-dimensional periodicity. For such aperiodic structures the standard crystallographic tools fail and, in order to remedy the problem, one usually resorts to the so-called superspace approach. Here, the aperiodic structure is embedded in a fictitious higher-dimensional space where it becomes periodic again and the established, of course properly adapted, crystallographic tools (symmetry groups, reflection conditions, structure factors, …) regain their power (see *e.g.* Janssen *et al.*, 2007 ▸; van Smaalen, 2007 ▸; Pinheiro & Abakumov, 2015 ▸). As far as denisovite is concerned, no clear evidence for the occurrence of satellite reflections has yet been found, in contrast with the closely related charoite for which corresponding observations have been reported (Rozhdest­venskaya *et al.*, 2010 ▸).

Most mineral structures are not composed of molecules as basic building units. Nevertheless, many mineral structures still lend themselves to a notional hierarchical decomposition, resulting in the identification of peculiar structural subunits or modules. These can have various dimensions, sizes and shapes, such as clusters, chains, tubes, slabs, layers or even blocks. These subunits usually carry electrical charges. For an important subset of modular structures, so-called order–disorder or OD structures, partial symmetry operations can be identified which greatly help in the determination, description and understanding of such structures and the closely related phenomenon of polytypism (for definitions of the terms ‘modular crystal structure’, ‘polytypism’ and ‘OD structure’, the reader is referred to the Online Dictionary of Crystallography, http://reference.iucr.org/dictionary; see also Ferraris *et al.*, 2004 ▸; Merlino, 1997 ▸). Referring again to denisovite, it will be shown further below that its structure lends itself to a description using modular building units. It further shows twinning and stacking faults on very short length scales resulting in strongly diffuse diffraction patterns, both observations being typical for polytypic structures. As a side note, we mention that polytypism and the occurrence of partial symmetries are not a privilege of mineral structures: many small molecular structures, organic as well as inorganic, are also polytypic structures, *e.g.* WO_2_Cl_2_ (Jarchow *et al.*, 1968 ▸), Pigment Red 170 (Warshamanage *et al.*, 2014 ▸) or quinacridone (Gorelik *et al.*, 2016 ▸), and partial or non-crystallographic symmetry operations also occur amongst small molecular structures with *Z*′ > 1 (*cf*. Brock, 2016 ▸) and in macromolecular structures (see *e.g.* Mooers, 2016 ▸).

In contrast with well defined and controlled laboratory experiments, the conditions under which natural crystals grow are usually variable and often far from thermodynamic equilibrium. Parameters like temperature, pressure, elemental composition, pH, redox conditions *etc.* often change as a function of time and space. Many natural crystals are therefore inhomogeneous: they may exhibit concentration gradients, reaction rims or weathering crusts, fluid or solid inclusions, intergrowth with other minerals, cracks, fissures or etching. Since these characteristics accumulate (or disappear) over time, such crystals may be regarded as having registered information on the processes they have undergone throughout the whole period of their existence. From a practical point of view, this might imply that a studied natural crystal is often an individual rather than a faithful representative of all crystals of a given mineral species.

Most, if not all, of the above-mentioned particularities of mineral structures meet in denisovite. Furthermore, it forms assemblages or possibly even intergrowths with a number of other minerals from which it is difficult to isolate in pure form. To make things worse, denisovite does not occur in the form of regular crystals, but as fibres of only 200–500 nm in diameter. These fibres may reach lengths of more than 100 µm, but they are generally bent and cannot be considered as single crystals. This habit is unsuitable for a structure determination by standard single-crystal methods and probably explains why the structure, symmetry, chemical composition and even lattice parameters of denisovite have remained only approximately known until the present work, despite its official approval as a new mineral species in 1984. In particular, recent advances in the field of electron crystallography have enabled the study of single nanocrystals down to 5 nm in diameter, by enabling the collection of diffraction data and the solution of their structures by combinations of nanoscale electron diffraction methods. We have successfully used some of these new tools to determine the structure of the related mineral charoite with formula (K,Sr,Ba,Mn)_15–16_(Ca,Na)_32_[(Si_70_(O,OH)_180_)](OH,F)_4.0_·*n*H_2_O (Rozhdestvenskaya *et al.*, 2010 ▸, 2011 ▸), which is also only present as microsized fibres. Since the monoclinic symmetry and lattice parameters of charoite are very similar to those of denisovite, we decided to meet the challenge and try to solve the structure of denisovite as well.

Unlike charoite, all examined nanocrystals of denisovite exhibited an extremely high degree of diffuse scattering, which prevented us from following the same procedure as for charoite and forced us to make a detour. The successful structure solution employed a suite of complementary methods. We used electron microprobe analysis to determine the chemical composition, X-ray powder diffraction (XRPD) for the refinement of lattice parameters, and several state-of-the-art methods of electron crystallography, *viz*. transmission electron microscopy (TEM), selected-area electron diffraction (SAED), high-resolution transmission electron microscopy (HRTEM) and high-angle annular dark-field imaging (HAADF). These investigations, and previous experience with related minerals, allowed a preliminary structural model to be constructed, which in the end turned out to be already almost correct. At a later stage, an extensive search for a less-disordered nanocrystal of denisovite was successful and enabled us to collect three-dimensional diffraction data using electron diffraction tomography (EDT) (Kolb *et al.*, 2007 ▸, 2008 ▸) in combination with precession electron diffraction (PED) (Vincent & Midgley, 1994 ▸; Own, 2005 ▸; Avilov *et al.*, 2007 ▸; Mugnaioli *et al.*, 2009 ▸). From this, the space group of denisovite could be determined, intensities extracted and processed, and the structure solved *ab initio* and refined, including occupancies for mixed positions. At the final stage, the correctness of the structure solution was confirmed by Rietveld refinement against XRPD data obtained from an almost-pure powder sample. This final confirmation was particularly important, given the problematic characteristics of the denisovite crystals and assuming that only averaging over many individual crystallites would provide a statistically meaningful result. On the other hand, very probably the structure could not have been determined using XRPD methods alone. The synergistic relationship between electron crystallography and XRPD has recently been noticed and its importance emphasized by several authors (Yun *et al.*, 2015 ▸; Hao, 2015 ▸; Palatinus, 2015 ▸; Batuk *et al.*, 2015 ▸; McCusker & Baerlocher, 2013 ▸, 2009 ▸; Gorelik *et al.*, 2009 ▸). From the results of the structure determination and refinement, the details of the structure and its modules could be elucidated, including the positions of the intra-channel species. Altogether, this has allowed us to discuss important topological aspects, to deduce the crystal-chemical formula, and to explain the OD character of denisovite and thereby the origin of the diffuse scattering.

## 2. Denisovite, its occurrence and early investigations   

Denisovite is a rare mineral as it is found at only two localities, *viz*. at the Eveslogchorr and nearby Yukspor Mountains in the Khibini massif, Kola Peninsula (Menshikov, 1984 ▸), and later on in the Murun massif, Yakutia (Konev *et al.*, 1987 ▸), both in Russia. Both localities are linked to alkali-rich intrusions, and the formation of the mineral is probably due to hydrothermal or metasomatic alteration of parent rocks. Despite its global rarity, denisovite is relatively abundant at both localities. It occurs as acicular aggregates of greyish colour and is closely associated with nepheline, potassic feldspar, aegirine, fluorite, apatite, biotite and yuksporite in the Khibinis (Menshikov, 1984 ▸), and with aegirine, kalsilite and feldspar in the Murun massif (Konev *et al.*, 1987 ▸, 1996 ▸). Photographs of denisovite can be found online, *e.g.* at https://www.mindat.org.

Some basic properties of denisovite were described by Menshikov (1984 ▸). Based on wet chemical analysis he proposed as a preliminary crystal chemical formula (Ca_3.90_Mn_0.09_Sr_0.02_)(K_1.35_Na_0.63_Rb_0.01_)Si_6_O_16_(F_1.07_(OH)_0.93_), idealized as Ca_4_(K_1.4_Na_0.6_)Si_6_O_16_(F,OH)_2_, and from XRPD he found monoclinic symmetry and approximate lattice parameters *a* = 30.92, *b* = 7.2 and *c* = 18.27 Å, β = 96.3° and *Z* = 10. In 1984 denisovite was approved as a new mineral by the CNMMN (Commission on New Minerals and Mineral Names, since 2006 Commission on New Minerals, Nomenclature and Classification, CNMNC) of the IMA, the International Mineralogical Association. Based on certain similarities, Menshikov (1984 ▸) suggested that denisovite might be structurally related to xonotlite, Ca_6_(Si_6_O_17_)(OH)_2_, and charoite. Both minerals occur in the Murun massif, and in fact for charoite this is the only known locality. Charoite, (K,Sr)_15–16_(Ca,Na)_32_[Si_6_O_11_(O,OH)_6_]_2_[Si_12_O_18_(O,OH)_12_]_2_[Si_17_O_25_(O,OH)_18_]_2_(OH,F)_4_·∼3H_2_O, occurs in association with other alkali calcium silicates like frankamenite, K_3_Na_3_Ca_5_(Si_12_O_30_)(F,OH)_4_·H_2_O (Nikishova *et al.*, 1996 ▸), canasite, K_3_Na_3_Ca_5_Si_12_O_30_(OH)_4_ (Dorfman *et al.*, 1959 ▸), and miserite, K_1.5−*x*_(Ca,Y,*REE*)_5_(Si_6_O_15_)(Si_2_O_7_)(OH,F)_2_·*y*H_2_O (*REE* = rare earth elements; Schaller, 1950 ▸). The formulae given for these five minerals are idealized and are taken from the official IMA–CNMNC list, http://nrmima.nrm.se. The structures of all these minerals contain dreier tubular or dreier double silicate chains [‘dreier’ denotes, according to the nomenclature of Liebau (1985 ▸), chains of corner-linked SiO_4_ tetrahedra with a repeat unit of three]. As a consequence, all have a lattice parameter of about 7.2 Å along the chain axis. This value is characteristic of dreier silicate chains linked to edge-connected (Ca,Na)(O,OH)_6_ octahedra *via* common O atoms, whereby the repeat unit of three SiO_4_ tetrahedra matches the edge lengths of two octahedra. This 3:2 relationship results in an ambiguity, because the linkage can happen at two distinct sites about 3.6 Å apart. In addition to the minerals listed above, this structural motif is also well known from the wollastonite group, Ca_3_[Si_3_O_9_], from the tobermorite group, idealized as [Ca_4_Si_6_O_17_·2H_2_O]·(Ca·3H_2_O), and from the so-called C-S-H phases (calcium silicate hydrates). C-S-H phases are a group of synthetic minerals which crystallize upon the hydration and setting of Portland cement, which gives them outstanding technical importance (Bonaccorsi & Merlino, 2005 ▸).

From the similarity of the preliminary lattice parameters of denisovite with those of charoite, and from the chemical composition, it might be assumed with reasonable certainty that denisovite also belongs to the family of alkali-bearing calcium silicates with dreier tubular silicate chains, and that its structure is composed of building modules similar to those which occur in charoite (Rozhdestvenskaya *et al.*, 2010 ▸, 2011 ▸), frankamenite, canasite or miserite (Rozhdestvenskaya & Nikishova, 2002 ▸).

## 3. Experimental   

### 3.1. Microprobe analysis   

The elemental composition was determined by microprobe analysis using a wavelength-dispersive Link AN-10000 on an automated CamScan 4-DV electron microprobe at the V. G. Khlopin Radium Institute (analyst Yu. L. Kretser). The conditions of the experiment were as follows: accelerating voltage 20 kV, beam current 4 nA, data-collection time 60 s (excluding dead time). The following standards for *K*α X-ray lines were used: Na albite, K orthoclase, Ca diopside, Si almandine, Al kyanite, Fe pure iron and Mn manganese. Calculations of the preliminary crystal-chemical formula on the basis of microprobe analysis data were carried out on the basis of a total of six cations.

### 3.2. Preliminary XRPD   

An initial XRPD pattern of denisovite was obtained at the X-ray Diffraction Center of Saint Petersburg State University using a Rigaku Ultima IV diffractometer, 40 kV, 30 mA, angle interval 3° < 2θ < 100° with Δ2θ = 0.02°, scan speed 0.25° min^−1^, graphite monochromator, Cu *K*α_1_ radiation (λ = 1.5406 Å). Data processing, cell-parameter determination and qualitative phase recognition were performed using the program *PDWIN* (Fundamensky & Firsova, 2009 ▸). Peak parameters were refined by peak profile fitting. Refined parameters included the angular position and peak intensity of the α_1_ component, FWHM, profile asymmetry and shape factor. Calculations of unit-cell parameters were performed with 119 reflections in the interval 4.2° < 2θ < 100° with Δ2θ = |2θ_exp_ − 2θ_calc_| < 0.01°, and relative intensity (*I*/*I*_0_)_exp_ ≥ 1 (where *I*_0_ is the highest intensity, set as 100). Qualitative phase recognition, performed on the basis of 2θ angular positions and the intensities of the strongest lines, revealed that the sample contained significant amounts of impurities, mostly sanidine, orthoclase, nepheline and kalsilite.

### 3.3. TEM studies   

Samples for TEM studies were prepared by crushing in an agate mortar and dispersing the fibrous fragments on holey carbon films supported by copper grids. They were studied by conventional and high-resolution TEM and SAED using Philips CM20 and Tecnai F30 microscopes operated at 100 and 200 kV, respectively.

For HAADF-STEM (high-angle annular dark-field scanning transmission electron microscopy) an electron-transparent TEM lamella of about 50 nm thickness was prepared using an FEI dual-beam Nova focused ion beam (FIB) facility, a Ga ion beam acceleration voltage of 30 kV and beam currents from 20 nA to 30 pA. Subsequently, amorphous surface layers were removed by argon ion etching in a Gentle Mill from TechnOrg Linda at 400 V for about 10 min at a 15° gun angle. The sample was investigated in a non-probe-corrected FEI Titan 80-300 ST operated at 300 kV (spherical aberration constant of 1.2 mm) using spot size 9, condenser 2 and an aperture of 50 µm diameter, yielding a semi-convergence angle of about 8.8 mrad. Using this setting a spatial resolution of about 1.2 Å was achieved. The camera length was set to 196 mm, which allowed the collection of electrons scattered to angles of about 36–230 mrad using a Fischione annular dark-field detector. Denisovite was so beam-sensitive that fast operation was necessary, despite the small beam current of less than 10 pA. Noisy contrast due to this fast scanning was removed by slight Wiener filtering.

Image simulations within the frozen lattice approach using ten configurations were carried out by the *STEMSIM* software (Rosenauer & Schowalter, 2007 ▸). We used the probe and detector conditions as indicated above. Phase gratings were computed for patches of 936 × 1120 pixels up to a thickness of about 10 nm. The unit cell of denisovite was scanned using 93 × 155 probe positions at a defocus value of −48 nm, roughly corresponding to Scherzer conditions. The finite size of the source was taken into account by a convolution with a Gaussian of width 80 pm.

For EDT and PED studies, the sample was powdered in an agate mortar, suspended in ethanol, ultrasonicated and piped onto a Cu half-mesh. Experiments were performed employing a JEOL GEM 2010 TEM with an LaB_6_ electron source, an acceleration voltage of 300 kV and UHR pole pieces. A JEOL EM-21340HTR high-tilt specimen retainer was used in order to reach a tilt range of up to ±55°. EDT data were collected in steps of 1° in SAED-PED mode. PED was performed by a NanoMEGAS Spinning Star device with a precession angle of 1°. Diffraction patterns were acquired with an Olympus Tengra CCD camera (14-bit, 2048 × 2048 pixels).

**Figure 1:**
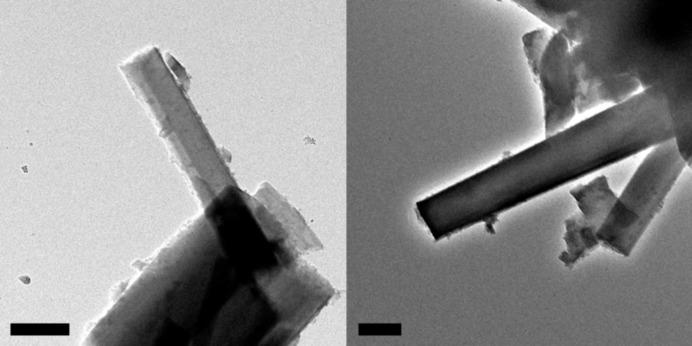
TEM images of crushed denisovite crystal fibres used for EDT data acquisition (namely crystal 1 and crystal 3). The scale bar is 200 nm.

Three EDT data sets were collected from three different fibres with respective thicknesses of about 160, 200 and 230 nm (Fig. 1 ▸). EDT data were elaborated and analysed with the *ADT3D* software (Kolb *et al.*, 2008 ▸; Mugnaioli *et al.*, 2009 ▸; Kolb *et al.*, 2011 ▸; Schlitt *et al.*, 2012 ▸) and routines developed in-house. Reflection intensities were extracted separately for each of the three data sets. For the diffuse scattering rods, intensities were just sampled where the reflection should be according to the determined unit cell. Structure solution was performed independently for the different data sets, systematically testing different resolution cuts. Combinations of two or more data sets were not considered. Only the best structure solution was taken for the final structure refinement.

The *ab initio* structure solution was performed by direct methods as implemented in *SIR2014* (Burla *et al.*, 2015 ▸) using a fully kinematic approach, *i.e.* intensities were assumed to be proportional to *F_hkl_* ^2^. A preliminary least-squares structure refinement was performed using *SHELXL97* (Sheldrick, 2015 ▸). Soft restraints (SADI) were imposed on Si—O and O—O distances. The least-squares refinement of Ca and K site occupancies, cation coordinates and atomic displacement parameters was performed using *WinCSD* (Akselrud & Grin, 2014 ▸). Electron scattering factors for the refinement were extracted from the *SIR* database. For the final refinement, cell parameters from XRPD were used.

### 3.4. Rietveld refinement   

A denisovite sample that had previously been shown to have a very low level of impurities was selected for the Rietveld refinement. The X-ray powder diffractogram was measured on a Stoe Stadi-P diffractometer equipped with a Ge(111) monochromator and a linear position-sensitive detector, using Cu *K*α_1_ radiation (λ = 1.5406 Å). The denis­ovite fibres were ground in a mortar with the addition of about 10% cork powder, in order to reduce the preferred orientation. The mixture was filled into glass capillaries, which were spun during the measurement. The 2θ range was 3 < 2θ < 100° with a total time for data acquisition of 110 h.

Rietveld refinements were performed with the program *TOPAS* (Coelho, 2007 ▸). The refinements started from the crystal structure determined by EDT. All atomic coordinates were refined, together with lattice parameters, peak profile parameters, scale factor and background. Additionally, the occupancies of the K^+^ ion positions could be refined. The remaining preferred orientation was modelled by spherical harmonics. A sensible geometry of the silicate framework was ensured by restraints on Si—O bond lengths and O—Si—O bond angles. The data quality did not allow refinement of the Ca/Na occupancies nor of atomic displacement parameters.

For control purposes, a Rietveld refinement was carried out without any correction for preferred orientation. All other settings were left unchanged. The resulting fit was worse, but the structure maintained almost unchanged (see §4.3 and the supporting information for more details).

### 3.5. Vibrational spectroscopy (discarded)   

At an advanced stage of the process of structure determination it was considered whether it might be useful to employ IR or Raman spectroscopy in order to obtain some definitive information on the OH^−^ groups. However, this idea was rapidly dropped, given the simultaneous presence of water in the structure of denisovite and of barely avoidable water absorped on the very high surface area of the very thin fibres.

## 4. Results and discussion   

### 4.1. Bulk sample analysis   

The average chemical composition based on microprobe data (wt%) is: SiO_2_ 50.75, Al_2_O_3_ 0.76, FeO 0.77, MnO 1.03, CaO 30.49, Na_2_O 2.70, K_2_O 9.76, sum 96.26. The deviation from 100% is due to species which cannot be detected by microprobe, *viz.* H_2_O, OH^−^ and F^−^, but were found by wet chemistry (Menshikov, 1984 ▸). The elemental composition found for denisovite is typical for alkali calcium silicates. The main elements are Si, Ca, Na, K, O, H and F. Minor contents of Mn, Fe and Al were also found in some of these minerals. Following the calculation of Menshikov (1984 ▸), and without any further knowledge of the structure, the empirical chemical formula of denisovite could be written on the basis of six cations as K_0.72_(Ca_1.90_Na_0.30_Mn_0.05_Fe_0.04_)(Si_2.94_Al_0.05_)O_8_(F,OH)_1_, *Z* = 20, or as K_14.40_(Ca_38_Na_6_Mn_1_Fe_0.80_)_Σ=45.8_(Si_58.80_Al_1.0_)O_160_(F,OH)_20_, *Z* = 1. This formula is similar to that obtained by wet chemical analysis (Menshikov, 1984 ▸), but our electron microprobe analysis did not reveal any significant amount of Sr or Rb. Also, one should keep in mind that such measurements can always be partially biased by sample water absorption. More importantly, we assigned Na together with Ca, and not with K, because Na and Ca tend to be iso­morphous in alkali calcium silicates, whereas Na and K do not. Menshikov’s empirical formula was calculated on the basis of a total of six cations corresponding to the dreier single chain silicate pectolite, NaCa_2_(Si_3_O_8_)OH. However, this approach is dissatisfying, since in pectolite Na is not octahedrally coordinated, and thus Ca and Na are not isomorphous in this mineral. Furthermore, as discussed in more detail below, the denisovite structure consists of more complex silicate anions than that of pectolite. Since the K sites in denisovite are only partially occupied, and thus the K content is variable, the calculation of the empirical chemical formula of denisovite has to be based on a total of 27 cations, *viz.* 12 Ca plus 15 Si sites. In doing so the empirical chemical formula of denisovite becomes K_14.76_(Ca_38.76_Na_6.20_Mn_1.04_Fe_0.76_Al_1.08_)_Σ=47.84_Si_60.2_O_162_(F_16_O_2.06_OH_2.0_)·2H_2_O.

### 4.2. Unit cell, disorder and space-group ambiguity   

The XRPD pattern of denisovite is characterized by the apparent absence of reflections with *l* odd, and therefore the first set of low-angle reflections is of type *hk*0. Reflections with *l* even appear only for 2θ > 25°. We used the unit-cell setting from SAED and the parameters reported by Menshikov (1984 ▸) as starting values for indexing our XRPD pattern. The refinement of the lattice parameters leads to *a* = 31.024 (1), *b* = 19.554 (1) and *c* = 7.1441 (5) Å, β = 95.99 (3)° and *V* = 4310.1 (5) Å^3^. These parameters differ from those of Menshikov (1984 ▸) and Konev *et al.* (1987 ▸) in the choice of the unit cell, *i.e.* in our setting the *b* and *c* axes are interchanged and the monoclinic angle β is located between the long (*a*) and the short (*c*) unit axes.

**Figure 2:**
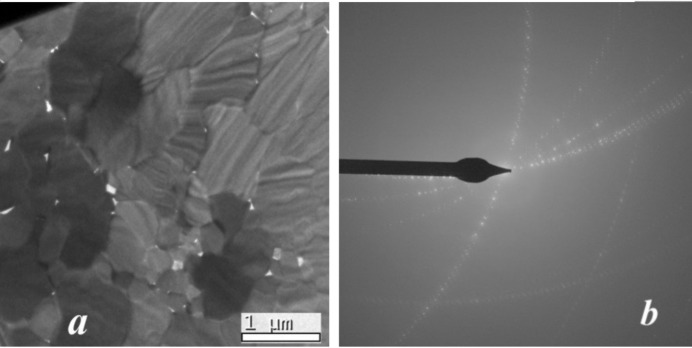
(*a*) Overview of an FIB prepared cross section (perpendicular to the *c* axis) of a fibrous denisovite sample. (*b*) SAED of an area containing four fibres. The sample is slightly tilted out of the zone-axis orientation for better visualization of the different orientations of the fibres.

**Figure 3:**
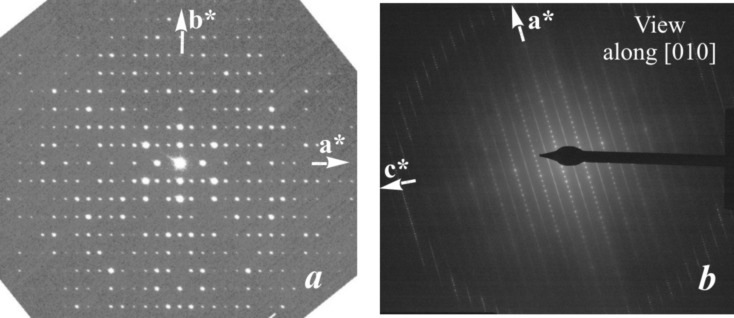
(*a*) SAED pattern along the *c* axis, showing that the a* and b* axes are mutually orthogonal. On the pattern, mirror symmetry exists along both axes and the reflection conditions *h*00: *h* = 2*n* are present. Weak violations of this rule are thought to be the result of residual dynamic effects. (*b*) SAED pattern along the *b* axis. In the pattern of the zero-order Laue zone (*h*0*l* reflections, inner circle) the reflections *h* = 2*n* + 1 are extinct in all reciprocal rows with *l* = 2*n*, while in the pattern of the first-order Laue zone (*h*1*l* reflections, outer ring) no systematic absences are observed.

The TEM image of the cross section of an acicular crystal aggregate of denisovite shows that it consists of single-crystal fibres with diameters typically around 200–500 nm (Fig. 2 ▸ *a*), having nearly parallel axes but different azimuthal orientations (Fig. 2 ▸ *b*). The short *c* axis runs along the fibre axes. The SAED pattern recorded along the *c* axis shows that a* and b* are perpendicular to each other (Fig. 3 ▸ *a*). Mirror symmetry appears on both axes and along a* the reflection conditions are *h*00: *h* = 2*n*. Weak violations of this rule, *e.g.* 900, are considered to be the result of residual dynamic effects. In the [010] SAED pattern (Fig. 3 ▸ *b*) the *h*0*l* zone reflections (inner circle) clearly show that, for all reciprocal rows with *l* = 2*n*, the reflections *h* = 2*n* + 1 are extinct, while they are present in the *h*1*l* zone (outer ring, Fig. 3 ▸ *b*). Fig. 3 ▸(*b*) also demonstrates that all *l* = 2*n* + 1 reciprocal rows are unstructured continuous diffuse rods running along a*. In view of the small size of the illuminated sample area, this indicates that structural disorder along the *a* axis must exist already on the nanometre scale. The strong diffuseness of all *l* = 2*n* + 1 reflections also explains why such reflections could not be observed in the XRPD pattern and it prevents us from making statements about the reflection conditions in these rows. Therefore, this leaves us with an ambiguity about the space group, as it could be either *P*12/*a*1 or *P*12/*n*1, provided the structure is centrosymmetric. Note, however, that both space-group symbols correspond to different cell choices of the same space-group type (standard setting *P*12/*c*1).

### 4.3. Direct-space approach: structural model of denisovite on the basis of the HAADF images   

**Figure 4:**
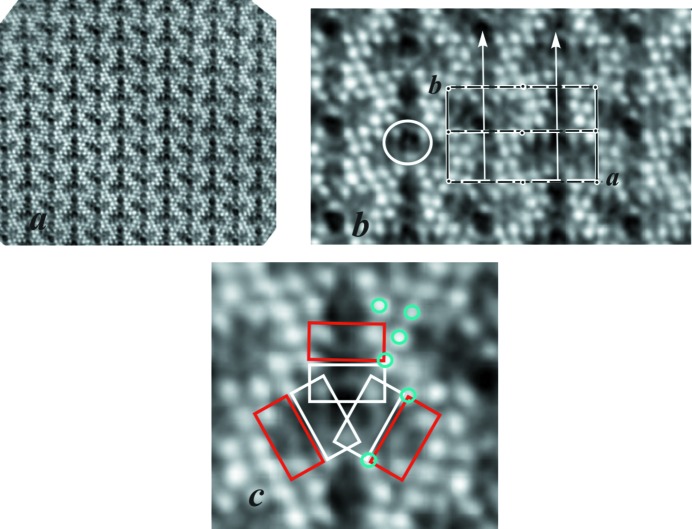
(*a*) Filtered [001] HAADF image of denisovite. (*b*) Enlarged part with the unit cell and symmetry elements; one of the circular contrasts is marked by a white circle. (*c*) Three weak arch-like fragments (red rectangles) are situated close to the three weak arch-like areas of the circular contrast (white rectangles). Turquoise circles indicate independent Ca positions.

In HAADF imaging the intensity of the scattered electrons is roughly proportional to the square of the atomic number of the scattering atoms. Therefore, heavier atoms result in stronger contrast and in favourable cases they can be distinguished from lighter atoms (*Z*-contrast imaging). Because the image produced is a projection of the investigated structure, it is preferably taken along a direction with short lattice spacing. In an early attempt to determine the structure of the related mineral charoite, a model could be developed just by interpretation of HRTEM and HAADF images combined with previous knowledge of the related minerals frankamenite and miserite (Rozhdestvenskaya *et al.*, 2009 ▸). The essential correctness of the model could be demonstrated when the newly developed EDT method subsequently became available, allowing *ab initio* structure solution (Rozhdestvenskaya *et al.*, 2010 ▸, 2011 ▸). In our study of denisovite, the high degree of disorder present in all examined nanocrystals caused us to suspect that an EDT approach would not be promising. Instead, the successful application of imaging methods to charoite convinced us to follow a similar approach for denis­ovite. The HAADF image (Fig. 4 ▸ *a*) was taken along the fibre axis, *i.e.* the short *c* axis. It shows, as expected, that the *a* and *b* axes are at right angles. The contrast in the unit cell, *i.e.* the projection along [001], shows a twofold rotation point, a mirror line perpendicular to *a* and a glide line perpendicular to *b*. Since the crystal system is monoclinic with unique axis *b*, the rotation point corresponds to an inversion centre, the mirror line to a twofold axis along the *b* axis and the glide line to a glide symmetry plane, either *a* or *n*, perpendicular to the *b* axis, as indicated in Fig. 4 ▸(*b*). Both possibilities, *a* or *n*, are indistinguishable in this projection and the observations are in accordance with the proposed possible space groups, *P*12/*a*1 or *P*12/*n*1, both of which have *p*2*mg* symmetry of their special projection along [001] (Hahn, 2005 ▸).

In order to compare the structures of denisovite and charo­ite, the respective HAADF images taken along the *c* axis are shown in Figs. 4 ▸ and 5 ▸. The charoite-96 polytype has lattice parameters *a* = 32.11 (6), *b* = 19.77 (4) and *c* = 7.23 (1) Å, β = 95.85 (9)° and *V* = 4565 (24) Å^3^ (Rozhdestvenskaya *et al.*, 2011 ▸), very similar to those of denisovite, and space group *P*12_1_/*m*1. Despite the metrical coincidence and general kinship of their structures, inspection of their HAADF images clearly demonstrates that both structures differ in their contrast and thus in the number and kind of silicate chains and in the mutual arrangement of silicate chains and octahedra walls.

**Figure 5:**
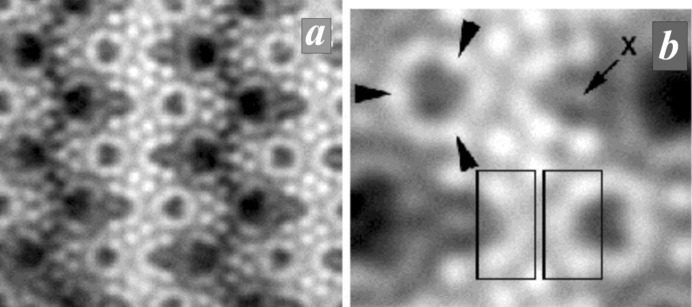
[001] HAADF-STEM images of charoite: (*a*) An overview. (*b*) Enlarged part of panel (*a*). The circular contrast has three stronger spots marked by arrowheads. The arch-like contrast has intense spots at both ends (left rectangle) and resembles part of the circular contrast (right rectangle). It is thought that the arch-like unit consists of two pectolite chains forming xonotlite dreier double chains with formula [Si_6_O_17_]^10−^.

**Figure 6:**
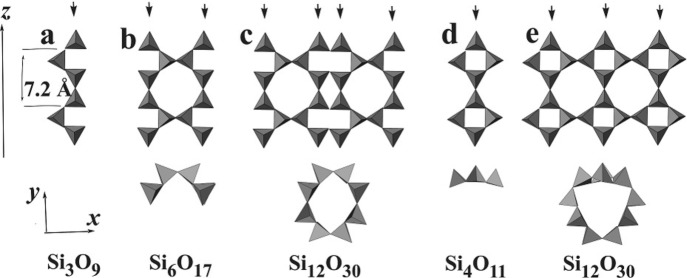
Dreier silicate chains, (top) unfolded and (bottom) viewed along the chain direction. (*a*) Dreier single tetrahedra chain (pectolite-like). (*b*) Dreier double chain (xonotlite-like). (*c*) Unfolded drawing of the tubular dreier triple chain in frankamenite and miserite. (*d*) Loop-branched dreier single chain. (*e*) Unfolded tubular loop-branched dreier triple chain in charoite and denisovite. Rows of superimposed tetrahedra along the tubular axis are indicated by arrowheads. In panels (*b*), (*c*) and (*e*), eight-membered rings of tetrahedra exist, ‘8MR’.

A common feature of charoite, frankamenite and miserite is that their structures contain tubular silicon–oxygen chains with composition [Si_12_O_30_]^12−^ per unit translation along the *c* axis. In HAADF images of charoite (Rozhdestvenskaya *et al.*, 2009 ▸) these tubular chains appear as circular contrast (Fig. 5 ▸). Similar contrasts can be identified in the HAADF image of denisovite, one being marked by a white circle in Fig. 4 ▸(*b*). These relatively regular circles consist of three bright spots, with weaker arch-like intensities connecting them. Their shape and size leads us to hypothesize that the circular contrast is caused by the same type of tubular silicate chain, [Si_12_O_30_]^12−^, as found in charoite (Fig. 5 ▸ *b*). The more intense spots on the circle are interpreted as projections of rows of superimposed tetrahedra along the tubular axis. These rows are indicated by arrowheads in the schematic drawing of these unfolded chains in Fig. 6 ▸. The distribution of the three bright spots has twofold axial symmetry, thus two spots are related by the twofold axis and the third one coincides with its position, *i.e.* it occupies a special position with *x* = 0.25 (Fig. 4 ▸ *b*).

According to the classification of silicates (Liebau, 1985 ▸), the dreier single chain of pectolite (or wollastonite) (Fig. 6 ▸ *a*) can be regarded as the fundamental chain of a dreier double chain as in xonotlite (Fig. 6 ▸ *b*) and of the tubular dreier triple chains found in frankamenite and miserite (Fig. 6 ▸ *c*). By way of contrast, the fundamental chain of the tubular loop-branched dreier triple chain of charoite (*cf*. Figs. 9*b* and 9*e* of Rozhdestvenskaya *et al.*, 2010 ▸) and, as presumed at this stage, of denisovite, is a loop-branched dreier single chain (Figs. 6 ▸ *d* and 6 ▸ *e*). Loop-branched dreier single chains are also known from the structure of synthetic Li_2_Mg_2_[Si_4_O_11_] (Czank & Bissert, 1993 ▸).

In Fig. 4 ▸(*c*) one can further observe three arch-like contrasts, indicated by red rectangles, close to the three weak arch-like contrasts ascribed to the tubular chain. These arch-like fragments exhibit intense spots at both ends and resemble parts of the tubular chain (white rectangles in Fig. 4 ▸ *c*). From this observation, it can be supposed that these additional arch-like fragments consist of two pectolite chains and form a dreier double chain with formula [Si_6_O_17_]^10−^, as shown in Fig. 6 ▸(*b*) (*cf*. also, *e.g.*, Figs. 9*a* and 9*d* of Rozhdestvenskaya *et al.*, 2010 ▸). Such dreier double chains are also called xonotlite chains. So, assuming *P*12/*a*1 (or *P*12/*n*1) as the space group, it can be proposed that the unit cell of denisovite contains two tubular loop-branched dreier triple silicate chains with formula [Si_12_O_30_]^12−^ and six dreier double silicate chains with formula [Si_6_O_17_]^10−^.

Beside the features assigned to the silicate chains, the remaining intense spots in the unit cell can be assigned to (Ca,Na) octahedra. In the alkali calcium dreier chain silicates with period *c* ≃ 7.2 Å, the columns of edge-sharing octahedra have two polyhedra per unit translation along the *c* axis (*cf*. Figs. 11*a* and 11*c* of Rozhdestvenskaya *et al.*, 2010 ▸). Therefore, the six spots marked by blue circles in Fig. 4 ▸(*c*) correspond to 12 independent positions for (Ca,Na) octahedra.

The potassium cations can be located at the centre of the [Si_12_O_30_]^12−^ tubular chain and they also occupy positions close to the centres of the eight-membered tetrahedral rings which are formed within the tubular chains, as well as in the xonot­lite-like chains, as observed in related minerals (*e.g.* charoite, frankamenite, canasite and miserite). Some K^+^ positions may be only partially occupied.

**Figure 7:**
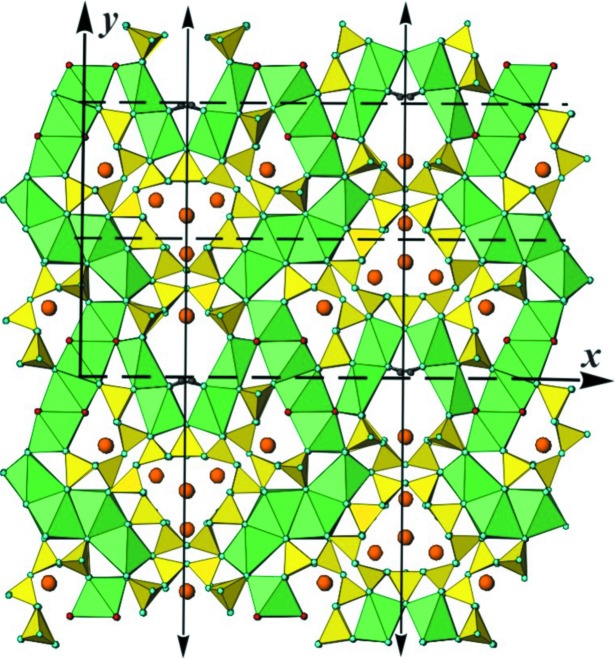
The structural model of denisovite based on HAADF image interpretation. The unit cell and symmetry elements are shown. Silicate chains are shown in yellow, (Ca,Na) octahedra in green and K^+^ ions as large brown circles, and turquoise, purple and black small circles represent oxygen, F/OH and H positions, respectively.

**Figure 8:**
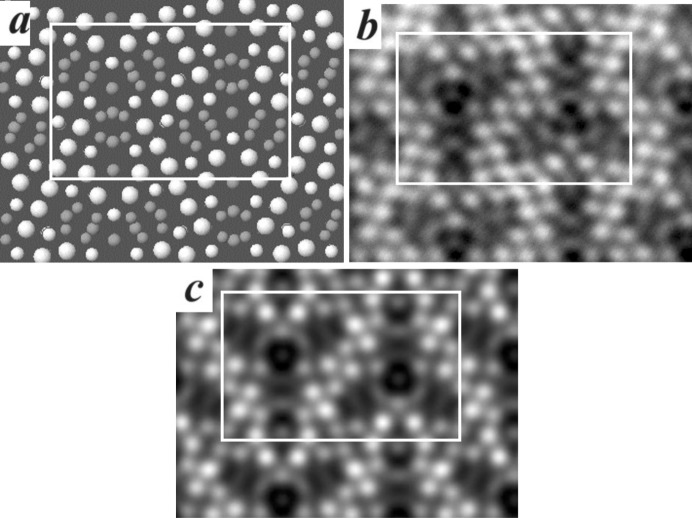
(*a*) The structure of denisovite projected along the *z* axis; only Ca, K and Si atoms are shown. Large white balls represent Ca atoms, small white balls represent Si atoms in vertical Si_2_O_7_ groups, and small grey balls represent Si atoms in horizontal Si_2_O_7_ groups and K atoms. (*b*) Experimental HAADF image of denisovite. (*c*) Simulated HAADF image calculated from the *ab initio* structure.

Based on these arguments, a structure model could be constructed and the general formula of denisovite expressed as K_14_(Ca,Na,Mn,Fe)_48_Si_60_O_162_(F,O,OH)_20_·2H_2_O, *Z* = 1 (Rozhdestvenskaya *et al.*, 2014 ▸). This model is shown in Fig. 7 ▸. Later it was shown that the model obtained by this direct-space approach was already in very good agreement with that determined *ab initio* (see next paragraph). The final structure, observed image and simulated high-resolution HAADF image, calculated from this structure, are compared in Figs. 8 ▸(*a*), 8 ▸(*b*) and 8 ▸(*c*), respectively.

### 4.4. Reciprocal-space approach: *ab initio* structure solution and refinement on the basis of EDT, PED and XRPD   

**Figure 9:**
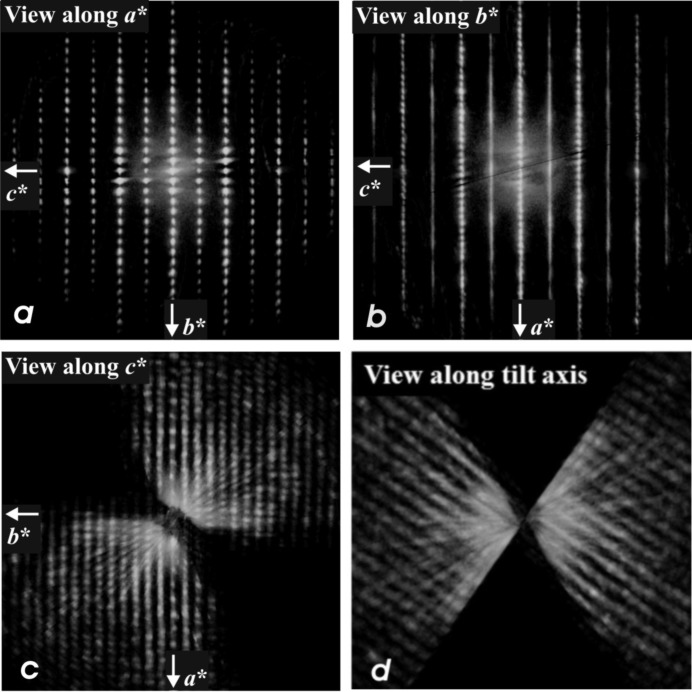
(*a*) EDT three-dimensional diffraction volume of denisovite viewed along a*. Reflections *hkl*: *l* = 2*n* + 1 are weaker but there is no evidence of diffuse scattering. (*b*) EDT three-dimensional diffraction volume of denisovite viewed along b*. Reflections *hkl*: *l* = 2*n* + 1 are weaker and show strong diffuse scattering along a*. (*c*) EDT three-dimensional diffraction volume of denisovite viewed along c*. (*d*) EDT three-dimensional diffraction volume of denisovite viewed along the tilt axis of the acquisition.

As reported above, the EDT method could only be performed with any real chance of success when an extensive search for a less disordered nanocrystal of denisovite was successful. The reconstructed EDT three-dimensional diffraction pattern showed that reflections *hkl*: *l* = 2*n* + 1 were significantly weaker than reflections *hkl*: *l* = 2*n* (Figs. 9 ▸ *a* and 9 ▸ *b*). The same feature was also observed for charoite and related minerals and indicates the half-periodicity of the octahedra walls (about 3.6 Å). Additionally, reflections *hkl*: *l* = 2*n* + 1 exhibit strong diffuse rods parallel to a* (Figs. 3 ▸ *b*, 9 ▸ *b* and 9 ▸ *c*), interpreted as heavy disorder already present on the nanometre scale. Remarkably, in charoite the same direction is affected by order–disorder (OD) stacking (Rozhdestvenskaya *et al.*, 2010 ▸, 2011 ▸). No diffuse scattering is observed along b* or c* (Figs. 3 ▸ *a* and 9 ▸ *a*). Because of the strong diffuse scattering along a* (Fig. 3 ▸ *b*), the reflection conditions for reflections *h*0*l* cannot be properly determined. Therefore, also from these data it cannot be decided whether the space group of denis­ovite is *P*12/*a*1 or *P*12/*n*1 (or even *P*1*a*1 or *P*1*n*1, if non-centrosymmetric).

For a diffraction pattern which exhibits such strong diffuse scattering, at least three different approaches for structure determination can be envisaged:

(i) Non-consideration of the diffuse reflections. If only the sharp Bragg peaks are taken into account, the average structure is obtained. Generally, the resulting structure model is strongly disordered and contains a superposition of different atomic positions. For denisovite such an approach would result in a model with *c*′ = *c*/2 ≃ 3.6 Å, consisting of a superposition of the atomic positions from the two halves of the original unit cell with *c* ≃ 7.2 Å. Compared with the HAADF approach, which had already provided a reasonable structural model with *c* ≃ 7.2 Å, non-consideration of the diffuse reflections would therefore result in a serious drawback.

(ii) Extraction of intensities at Bragg positions only. The extraction of Bragg intensities from a continuous diffuse rod is challenging and the resulting intensities may depend strongly on the algorithm used. However, in favourable cases this approach will allow for correct phasing of reflections.

(iii) Full analysis of the diffuse scattering. In this approach the intensity distribution along each diffuse rod must be measured carefully with high resolution. The structure must then be fitted simultaneously to both the Bragg peaks and the diffuse intensity. The resulting structural model reproduces the full diffraction pattern and contains information on the average structure as well as on local deviations thereof, such as stacking probabilities, local ordering, distribution of ions on mixed sites *etc*. For denisovite such an approach is barely possible, because the long *a* axis (∼31 Å) results in very short distances between neighbouring Bragg positions along a*, so that the intensity profile along the diffuse rods cannot be determined with sufficient accuracy by electron diffraction.

Therefore, we used approach (ii), *viz.* extraction of intensities at Bragg positions only, for the structure determination of denisovite. Reflection intensities were extracted independently from three EDT data sets where we could detect some hints of intensity maxima along diffuse rods, using a monoclinic cell with β ≃ 96°. The structure solutions were performed independently with the different data sets, systematically testing different resolution cuts and space groups. The best combination was obtained from data set 3, resolution limit 1.2 Å and space group *P*12/*a*1. No attempt was made to combine two or more data sets. The unit-cell, data-acquisition and refinement parameters are given in Table 1 ▸.

The structure was solved by direct methods as implemented in *SIR2014* (Burla *et al.*, 2015 ▸), using a fully kinematic approach. From the best solution (*R* = 0.3282, 1715 reflections with *F* > 8.4, *U*_overall_ 0.029 Å^2^), 12 Ca, 15 Si, five K positions and 34 out of 48 (O + F) sites were correctly identified. The missing 14 (O + F) sites of the framework and two sites inside the channels (one H_2_O molecule and one K^+^ ion) were located according to geometric considerations and the model proposed earlier (Rozhdestvenskaya *et al.*, 2014 ▸).

Least-squares refinement using *SHELXL97* (Sheldrick, 2015 ▸) and *WinCSD* (Akselrud & Grin, 2014 ▸) with 2454 reflections *F*(*hkl*) > 4σ(*F*) converged to an unweighted residual *R*_1_^ED^ = 0.336 (ED indicates electron diffraction). The number of independent atomic positions is 82 and the number of free variables is 321. Refinement of the occupancies of Ca, K and *W*1 (water) sites, of atom coordinates and of isotropic displacement parameters was performed. In order to stabilize the refinement, the Si—O bond lengths were restrained to the range 1.53–1.73 Å. No significant residual potential was found in the difference Fourier map.

The high value of 0.336 for *R*_1_^ED^ deserves comment. For a standard refinement of a small structure with data collected on an X-ray diffractometer using a crystal of good quality and after proper data reduction, such a value would clearly be unacceptable. However, our electron crystallographic diffraction study on denisovite was affected by several unavoidable adverse effects which precluded a better refinement. Besides the complexity of the structure, the nanometre size of the crystal, beam damage, residual dynamic effects and the variable thickness of the sample during data acquisition, but missing absorption correction, one must also accept a considerable degree of experimental inaccuracy in the intensities, because, after all, the data were collected using a microscope not a diffractometer. Moreover, for denisovite the disorder is not a slight disturbance of an otherwise rather perfect structure, but is an inherent structural feature. The structure we have solved and refined is just an average or ideal one, which is ordered only on a very limited spatial scale smaller than the acquisition area, say some 100–200 Å in diameter.

Despite the very defective structure amplitudes, the structure solution was successful because the phasing worked out correctly, and the refinement also went well. The residual obtained by the least-squares refinement should not be taken at face value, but rather as a figure of merit that actually tells us that denisovite is full of planar defects. The ratio of crystalline/ordered repetitions *versus* stacking faults is expected to be slightly higher than 1, *i.e.* the disordered part is far from being irrelevant and, since we must ignore it, because our currently available techniques do not allow us to do better, we have to accept the high residual. Therefore, the refined primary structural parameters like atomic positions, and derived parameters like bond lengths, angles and bond-valence sums (BVS), clearly have lower precision and accuracy than similar parameters obtained from standard X-ray procedures. Notwithstanding these challenges, the data were of sufficient quality to reveal the essential topological and geometric features of the structure, and even to find sites with mixed occupancy of cations. The refined atomic coordinates, isotropic displacement parameters and site occupancies are given in Table S1 in the supporting information, and selected interatomic distances are shown in Table 2 ▸. BVS are reported in Table S2 in the supporting information.

**Figure 10:**
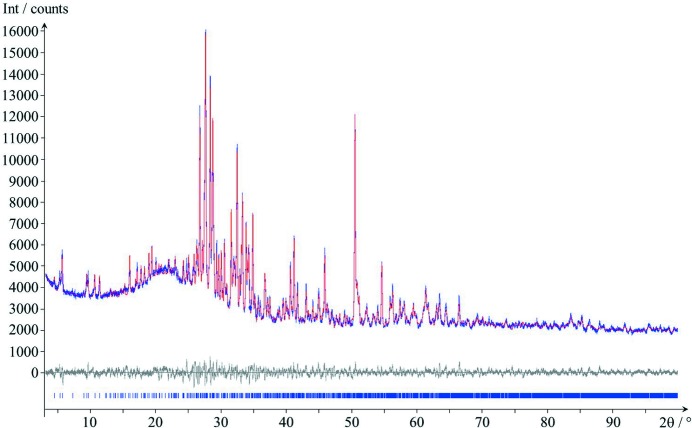
Rietveld plot. Experimental data are shown in blue, simulated data in red and the difference curve in grey. The blue vertical tick marks denote the reflection positions.

As stated above, EDT and XRPD methods are complementary and should therefore be performed in combination, whenever possible. This became possible when a powder sample of denisovite became available which was nearly free from impurities. Thus, the crystal structure was confirmed by Rietveld refinements. The space group used was *P*12/*a*1. The refinement of the lattice parameters results in similar values to those found from the initial powder data (see Table 1 ▸). All atomic coordinates were refined, as well as the occupancies of the K^+^ ions (Table S3 in the supporting information). The refinement converged to a low *R* value giving a rather smooth difference profile (Fig. 10 ▸). Crystallographic data are included in Table 1 ▸.

In agreement with what has already been observed in the three-dimensional EDT reconstructed diffraction volume, the visual inspection of the XRPD pattern shows that reflections with *l* = 2*n* are sharp and reflections with *l* = 2*n* + 1 are very broad. This explains some of the mismatch in the Rietveld plot, especially in the region between 13 < 2θ < 22° (Fig. S1 in the supporting information). A treatment of peak anisotropy with spherical harmonics, as is usually done by the *TOPAS* software, does not cover this situation, and consequently peaks *l* = 2*n* + 1 cannot be fitted properly.

A refinement without a preferred orientation correction was possible as well. The fit is slightly worse and the *R* values increase, but the structure does not change greatly (see Figs. S2 and S3 and Table S4 for details, in the supporting information).

**Figure 11:**
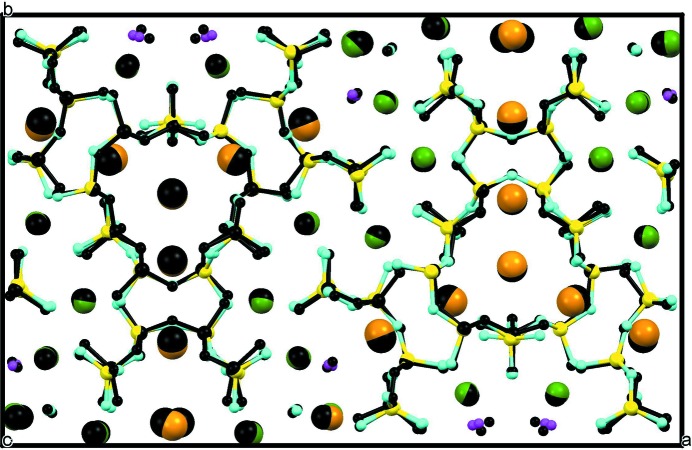
Superposition of the structures from EDT (coloured) and Rietveld refinement (black). Colour codes: Si yellow, O turquoise, Ca and Na green, K brown, and F purple.

The resulting structure is in very good agreement with that determined by EDT. A superposition of the two structures is shown in Fig. 11 ▸. The average and maximum atomic displacements between the structures refined on the basis of EDT and XRPD data are, respectively, 0.26 and 0.91 Å. The difference is smaller for heavy atoms (K, Ca, Na, Si), with average and maximum atomic displacements of 0.17 and 0.42 Å, respectively, and larger for light atoms (O, F), with average and maximum atomic displacements of 0.32 and 0.91 Å, respectively (comparison quantified by the *COMPSTRU* routine available at the Bilbao Crystallographic Server, http://www.cryst.ehu.es/).

## 5. Structure description and assignment of mixed-atom species   

The following discussion is based on the structure as determined and refined by EDT, since this solution is considered to be more accurate than those obtained from Rietveld refinement or HAADF. Whereas individual bond lengths (Table 2 ▸) and BVS (Table S2 in the supporting information) show considerable scatter, their mean values agree well with expectations. The mean BVS for Si is 4.11 (8) valence units (v.u.) with extremes of 4.23 and 3.94 v.u. The corresponding values for Ca are 2.07 (10) v.u., with a minimum of 1.90 and a maximum of 2.23 v.u. Partial substitution of Na for Ca has been neglected. On the basis of the BVS, two groups of O atoms can be distinguished. For atoms O1–O42 the mean BVS is 2.11 (26) v.u., with 2.75 and 1.65 v.u. as extremes. These positions are considered to be basically occupied by O^2−^. The group consisting of O43 and O44 has a mean BVS of 1.35 (2) v.u. These positions are therefore considered to be occupied by OH^−^ rather than O^2−^. The significant deviation from unity may be an indication of some degree of substitution of O^2−^ for OH^−^. Atoms F1–F4 have a mean BVS of 1.04 (28) v.u. The large standard deviation and the very high deviation from unity for F4 are probably the consequence of the fact that this atom has calculated interatomic distances from the disordered and weakly populated K6 site. The latter atom also contributes to the calculation of the mean BVS of K (0.95 v.u.), which is in fair agreement with the expected value but shows a large standard deviation and extreme scatter.

In general, the mean values for the interatomic distances agree well with expectation: the mean Si—O distance is 1.62 (5) Å, with minimum and maximum values of 1.53 and 1.73 Å, respectively. The corresponding values for Ca—O(1–42), Ca—O(43–44) and Ca—F are 2.36 (10)/2.62/2.18, 2.40 (14)/2.59/2.18 and 2.36 (13)/2.59/2.18 Å, respectively, and for K—O the mean is 3.09 (30) Å. Note that water has been neglected for the calculation of BVS and for the interatomic distances concerning Ca. Even though the mean values are reassuring, the precision of these data is significantly lower than what would be expected in the case of a standard diffraction experiment and therefore we will largely refrain from discussing individual bond lengths or even angles in detail, but rather focus on topological details, taking into account the just-mentioned BVS arguments.

The structure of denisovite contains 15 Si, 12 Ca, six K, 44 O, four F and one water positions, *i.e.* 82 independent atoms in its asymmetric unit (see Table S1 in the supporting information). Several positions have mixed or partial occupancies, not all of which could be refined or assigned with reasonable certainty. Four nominal Ca positions (Ca7, Ca8, Ca11 and Ca12) contain considerable amounts of Na, two nominal O positions (O43 and O44) are predominantly OH^−^ (see Table S2 in the supporting information), and some of the remaining O positions as well as the F positions may also contain some OH^−^. All Si and Ca atoms occupy the general Wyckoff position 4*g* of space group *P*12/*a*1, as do all the other atoms with the exception of seven atoms on special positions, *viz.* K1 on 2*e*, K4 and K5 on 2*f*, two O (O14 and O17) on 2*f* and one O (O1) on 2*e*, and water on 2*f*. All Si are tetrahedrally coordinated by four O^2−^ or OH^−^ and the (Ca,Na) are octahedrally coordinated by six species (O^2−^, OH^−^ and F^−^). The coordination of the K^+^ cations by O^2−^, OH^−^, F^−^ and water (*W*) varies between eight- and 13-fold, depending on the bond limits we set for the coordination (see Table 2 ▸).

**Figure 12:**
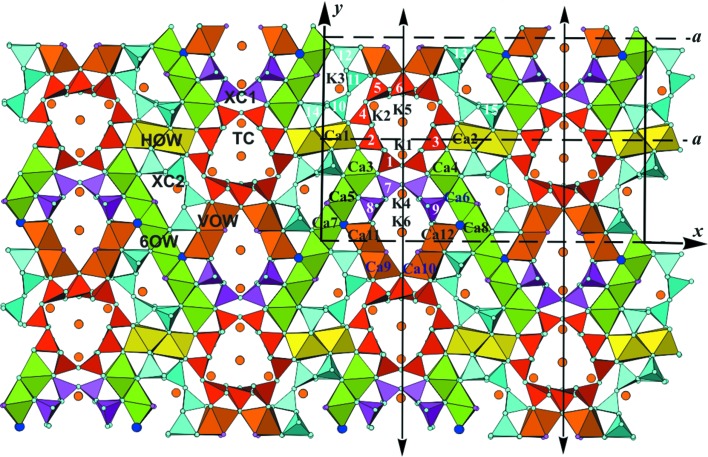
A polyhedral representation of the denisovite structure as obtained from the *ab initio* structure solution, shown down the *z* axis. The unit cell and symmetry elements are shown. Tubular loop-branched dreier triple chains [Si_12_O_30_]^12−^ (**TC**) are shown in red, and xonotlite-like dreier double chains [Si_6_O_17_]^10−^ (**XC1**) and (**XC2**) are shown in purple and turquoise, respectively; ‘horizontal’ octahedra walls (**HOW**) are coloured yellow, walls from two (Ca9—Ca12) octahedra (**VOW**) brown and six-octahedra-wide walls (**6OW**) green. Small turquoise circles are O atoms, purple circles are F atoms, O43 and O44 are marked by blue circles and large brown circles indicate K atoms. Si atoms are numbered.

Fig. 12 ▸ shows a projection of the structure along [001], with Si and (Ca,Na) coordinations represented by the respective polyhedra. K positions are indicated by large brown circles. We consider the polyhedra as primary building units and discuss their combination into secondary building units, or modules, and the subsequent mutual arrangement of the different modules to yield the entire modular structure.

### 5.1. The silicate modules   

**Figure 13:**
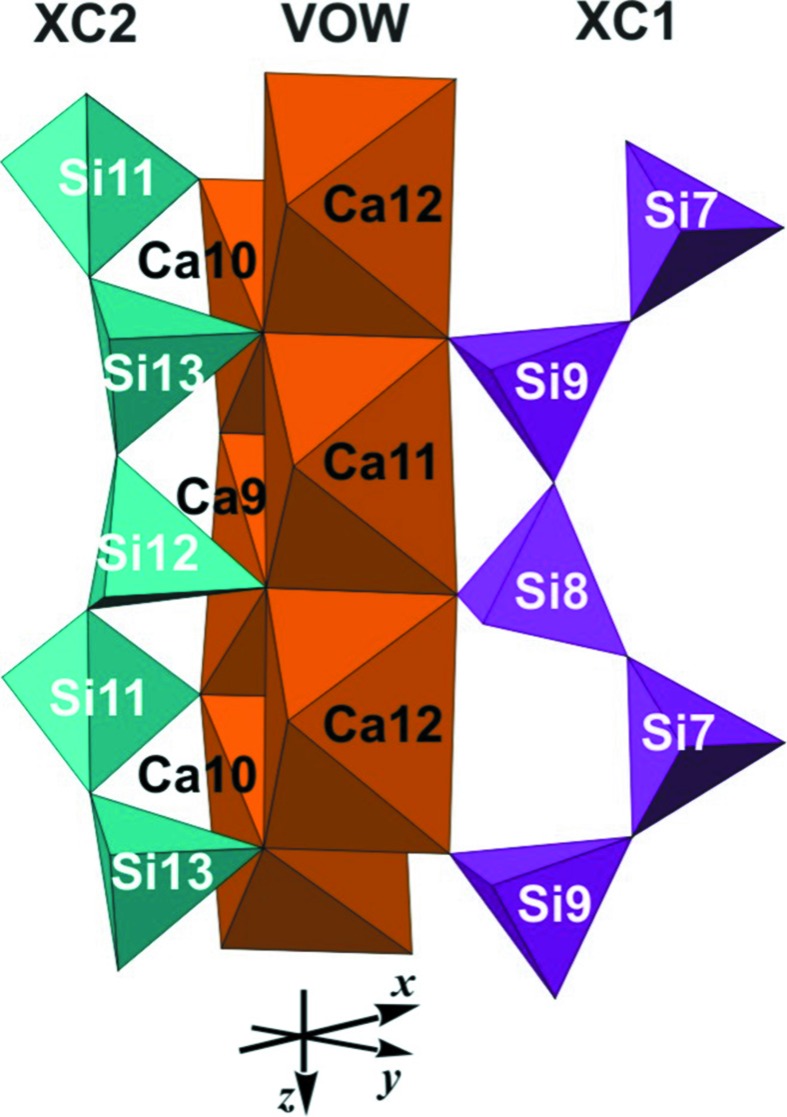
Denisovite, two strands of **XC2** (turquoise) and **XC1** (purple) on opposite sides of a **VOW**, showing that they are not displaced along *z* with respect to each other.

Two different types of silicate anion are easily distinguished, *viz.* bent dreier double chains, also called xonotlite-type chains, with composition [Si_6_O_17_]^10−^ in their repeat distance, and tubular loop-branched dreier triple chains, [Si_12_O_30_]^12−^. Unfolded representations of these different chains are shown in Figs. 6 ▸ *b* and 6 ▸ *e*, respectively (*cf*. also, *e.g.*, Figs. 9*a*, 9*b*, 9*d* and 9*e* of Rozhdestvenskaya *et al.*, 2010 ▸). All chains run along the *z* axis and are not directly connected to each other. The unit cell contains two symmetry-related tubular loop-branched dreier triple chains; for the sake of clarity these will be named **TC** henceforth, and they are coloured red in Fig. 12 ▸. In addition, it contains six xonotlite chains which can be subdivided into two distinct classes. Four of the xonotlite chains are related by the twofold axis and the glide plane; they are called **XC2** and are shown in turquoise. The remaining two xonotlite chains, called **XC1** and coloured purple, are mapped onto themselves by the twofold axis. **XC1** and **XC2** differ in the modules surrounding them, so they are symmetrically and topologically distinct. Perspective views of the silicate chains are given in Figs. 13 ▸, 14 ▸ and 15 ▸.

The **TC** has the same composition per repeat distance as the corresponding tubular chain in the mineral miserite, *viz.* [Si_12_O_30_]^12−^, however the fundamental chain in the latter is an unbranched dreier single chain, whereas that of **TC** is a loop-branched dreier single chain (Fig. 6*d* ▸). All SiO_4_ tetrahedra in the **TC** are connected *via* common corners to three more tetrahedra, *viz.* Si1 with (Si1′, Si2, Si3), Si2 with (Si1, Si3, Si4), Si3 with (Si1, Si2, Si4), Si4 with (Si2, Si3, Si5), Si5 with (Si4, Si6, Si6′) and Si6 with (Si5, Si5’, Si6′), with the primes indicating symmetry-related atoms. For each of the six independent Si in the **TC**, the three O atoms bridging between two tetrahedra (O_br_) and one non-bridging O atom, O_nb_ (separated from the former by a semicolon), are as follows: Si1 (O1, O3, O4; O2), Si2 (O3, O7, O8; O5), Si3 (O4, O7, O9; O6), Si4 (O8, O9, O11; O10), Si5 (O11, O12, O13; O16) and Si6 (O12, O13, O14; O15). All the O_nb_ in **TC** are bonded to two Ca atoms. O_br_ are charge-compensated, whereas O_nb_ either need additional coordination by K^+^ or might be partially replaced by OH^−^ in order to satisfy bond-valence requirements. With respect to the chain axis, differently oriented Si_2_O_7_ groups can be distinguished, *viz.* horizontal (Si1—Si1′, Si4—Si5, Si4′—Si5′) and vertical (Si2—Si3, Si3—Si2, Si6—Si6) ones. It is remarkable that all tetrahedra in **TC** are connected to three other tetrahedra; normally such **Q** ^3^ tetrahedra are characteristic of layer silicates. This fact lends itself to the suggestion that the **TC** can be regarded as a nano-scroll of pieces of a silicate layer (*cf*. Fig. 6*e* ▸). The same type of tubular chain has also been found in the structure of charoite (Rozhdest­venskaya *et al.*, 2010 ▸, 2011 ▸). A general classification and discussion of tubular chains occurring in silicate structures is given by Rozhdestvenskaya & Krivovichev (2011 ▸).

It has been mentioned earlier that the bent dreier double chains of composition [Si_6_O_17_]^10−^, **XC1** and **XC2**, are similar to those which occur in the structure of xonotlite. The tetrahedra of **XC1** are centred by atoms Si7–Si9. Si7 connects the single strands of the double chain and is connected to three neighbouring tetrahedra, centred by Si7′, Si8 and Si9. Si7 and Si7′ form a horizontal Si_2_O_7_ group. Thus, Si7 tetrahedra are of type **Q** ^3^, whereas Si8 and Si9 are **Q** ^2^. Within **XC1** there are four O_br_ (O17–O19, O25) which are charge-compensated, and five O_nb_ (O20–O24) which make additional bonds with two (O20, O23, O24) or three (O21, O22) Ca atoms.

**XC2** consists of SiO_4_ tetrahedra Si10–Si15. Si10 and Si11 are **Q** ^3^, forming horizontal Si_2_O_7_ groups, while the remaining Si atoms are **Q** ^2^ and form vertical Si_2_O_7_ groups. O atoms in these two chains are O26–O42, of which seven are O_br_ (O26, O29–O31, O36, O37, O42) and therefore make no bonds to Ca. The remaining ten (O27, O28, O32–O35, O38–O41) are O_nb_ and link the SiO_4_ tetrahedra to (Ca,Na) octahedra. They have either two (O27, O28) or three Ca bonding partners.

### 5.2. The octahedra modules   

The primary building units of the octahedron modules are CaO_6_ octahedra, with partial substitution of Na for Ca. Ca/Na isomorphism is typical for alkaline-bearing Ca silicates (Rozhdestvenskaya & Nikishova, 2002 ▸). The 12 symmetrically independent octahedra are distributed among six topologically different classes, each counting two octahedra. All octahedra are arranged in columns along the *z* direction. Topologically equivalent octahedra alternate along the columns, whereby each octahedron shares opposite edges with adjacent octahedra. These two opposite edges are roughly parallel to (001), *i.e.* the plane of drawing in Fig. 12 ▸. Their four corners define an approximate plane which will be referred to as the equatorial plane of the considered octahedron. The ‘upper’ two corners (with respect to the plane of drawing) of the equatorial plane will be denoted *u* or *u*′ where appropriate, the ‘lower’ ones *d* or *d*′. The corresponding octahedron edges can then be identified by the pairs of corners, such as *u*–*u*′, *d*–*d*′ or *u*–*d* *etc.* The remaining two corners of the octahedron will be called its apices and are denoted *a* or *a*′. Their position with respect to the *z* direction is approximately halfway between those of the corresponding *u*–*u*′ and *d*–*d*′ edges of the same octahedron.

Three different types of one-octahedron-thick sheets of edge-sharing octahedra are easily identified from Fig. 12 ▸. They extend periodically along the *z* direction with a repeat unit of two octahedra, corresponding to the *c* lattice parameter. Since these sheets are reminiscent of brick walls, we prefer to call them ‘walls’. In projection they appear as bands. The walls consist of parallel octahedra columns. The individual columns are linked *via* edge-sharing with either one or two neighbouring columns. In the first case all octahedra share four edges, in the second one six. Neighbouring columns are mutually displaced along [001] by *c*/4, or half the length of a vertical octahedron edge *u*–*d*. Because of symmetry, it makes no difference if the displacement is *c*/4 or −*c*/4.

In the following, CaO_6_ octahedra are identified by their respective Ca positions, indicated in Fig. 12 ▸. Two wall types have a width of two and one a width of six octahedra columns. One two-octahedra-wide wall consists of octahedra centred by Ca1 and Ca2, both with full Ca occupancy and topologically equivalent. It stretches roughly along [100] across an inversion centre. Since this octahedra wall is roughly horizontal in Fig. 12 ▸, it will be called **HOW** and is coloured yellow. The other two types of wall form symmetry-related pairs stretching roughly along [140] and [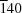]. These walls are two (Ca9–Ca12) or six octahedra wide (Ca3–Ca8). Although the orientation of the former is only roughly vertical to that of **HOW**, they are denoted **VOW**, and are coloured brown. Symmetry-related pairs of the **VOW** share common corners, thus forming V-shaped double walls. The six-octahedra-wide walls also stretch across inversion centres; they are denoted **6OW** and coloured green. Some of the octahedra in the latter two walls have partial substitution of Na for Ca, *viz.* Ca7, Ca8, Ca11 and Ca12 (*cf*. Table S1 in the supporting information). The imaginary continuations of all three types of octahedra wall intersect roughly at the centre of the **TC**. **HOW** and **6OW** share common corners and form infinite sequences …**HOW**–**6OW**–**HOW**–**6OW**… following zigzag paths along [010]. The double **VOW** share common corners with two adjacent **6OW**, thereby cross-linking neighbouring **HOW**/**6OW** sequences in the [100] direction.

The **HOW** are quite regular as both octahedra belong to the same topological class, and there is practically no Na substitution for Ca. Furthermore, all its corners are O^2−^ and connected to SiO_4_ tetrahedra. Both **VOW** and **6OW** are less regular. The octahedra columns of the **VOW** are topologically distinct. Ca9 and Ca10 have one vertical edge *u*–*d* whose apices are occupied by F^−^ (probably with some degree of substitution by OH^−^). These edges line up along [001] and their corners are marked by small purple circles in Fig. 12 ▸. The F positions are simultaneously apices *a* of the topologically equivalent Ca11 and Ca12 octahedra. The opposite apices *a*′ of the latter octahedra share corners with octahedra Ca7 and Ca8 of **6OW** at positions *u* and *d* and are alternately occupied by O43 and O44, marked by blue circles in Fig. 12 ▸. They also line up along [001] and are shared with three octahedra of the **6OW**, such that O43 and O44 are simultaneously *u* and *d* corners of Ca7 and Ca8, and apices *a* of Ca5 or Ca6, respectively. Neither O43 nor O44 is bonded to Si, thus both positions can be supposed to be occupied by OH^−^, because then O43 and O44 are nearly charge-compensated. This argument is in fact corroborated by BVS calculation (Table S2 in the supporting information). Note that both Ca7 and Ca8 octahedra have three corners *a*, *u*, *d* occupied by (F^−^/OH^−^), all of which are situated on one triangular side of the respective octahedron. This fact lends itself to the assumption that these octahedra should have a certain degree of Na-for-Ca substitution, which agrees well with the results of the structure refinement (56 and 33% Na, respectively, for Ca7 and Ca8). In **VOW** both octahedra Ca11 and Ca12 have both apices *a* and *a*′ occupied by F^−^ or OH^−^, respectively, and for these octahedra a certain degree of Na-for-Ca substitution could be expected too. Indeed, the refined occupancies were 22 and 40% Na, respectively. For the remaining octahedra in **VOW** and **6OW** no Na substitution could be found. The pairs of octahedra Ca3/Ca4, Ca5/Ca6 and Ca7/Ca8 of **6OW** form three different topological classes.

All octahedra in **VOW** are connected to SiO_4_ tetrahedra *via* those corners which are not occupied by F^−^ or OH^−^. For Ca9 and Ca10 these four corners are the apices *a* and *a*′ and the equatorial positions *u*′ and *d*′ opposite the line of F positions. For Ca11 and Ca12 all equatorial corners *u*, *u*′, *d* and *d*′ are shared with SiO_4_ tetrahedra, but not the apices *a* and *a*′. In **6OW** the terminal octahedra Ca3 and Ca4 are all corner-connected to SiO_4_ tetrahedra. Ca5 and Ca6 have three connections to SiO_4_ tetrahedra at those corners *a*, *u*, *d* which are not occupied by F^−^ or OH^−^ (*a*′, *u*′, *d*′); the same is true for Ca7 and Ca8.

It is important to emphasize that all the discussed OH^−^ and F^−^ positions, *i.e.* O43, O44, F1–F4, are not available for linkages to SiO_4_ tetrahedra. Thus, by virtue of an avoidance rule these positions can be suggested to play a certain structure-directing role.

### 5.3. Mutual arrangement of octahedra walls and silicate chains; explanation of the disorder   

Altogether the described octahedra walls form a rather rigid framework, with channels along [001] in which the various types of silicate anion are located. These channels are situated between zigzag sequences of **HOW** and **6OW** and form strips along [010]. The strips are sectioned by pairs of **VOW**. The **TC** are located within circular tubes formed by the front edges of, respectively, two **6OW**, **HOW** and **VOW**. The **XC1** are located in tunnels which in cross section resemble three-fingered leaves and are formed by two **6OW**, two **VOW** and a horizontal Si_2_O_7_ group (Si1—Si1′) of an adjacent **TC**. The **XC2** are located in ‘half-tubes’ formed by one **6OW**, one **HOW**, one **VOW** and one horizontal Si_2_O_7_ group (Si4—Si5) of an adjacent **TC**.

The silicate modules are geometrically constrained by their neighbouring octahedron walls, affecting both their conformation and their relative positions with respect to the octahedra framework. While the SiO_4_ tetrahedra are relatively rigid, the inter-tetrahedra angles Si—O—Si can vary over a wide range. Given the fact that, by linking SiO_4_ tetrahedra chains with CaO_6_ octahedra columns, the dimensions of the former have to match those of the latter, the silicate chains are forced to adopt a specific conformation in which an octa­hedron edge can be spanned either by O_nb_ of two neighbouring tetrahedra or by O_nb_ of a given and the next but one tetrahedron, whereby the intermediate tetrahedron is bonded to a neighbouring octahedra column (see Fig. 13 ▸). This leads to the typical kinked dreier silicate chain with a conformation described by torsion angles about Si⋯Si linkages in the sequence ⋯Si⋯Si′⋯Si′′⋯Si′′′⋯ alternating in the order of ∼180°, ∼180° and ∼0°.

Horizontal Si_2_O_7_ groups of a given **TC** and of the three adjacent **XC1** and **XC2** are bonded to vertical (*u*–*d*) octa­hedron edges of **HOW**, **VOW** or **6OW**, respectively, and are therefore displaced with respect to each other by the length of one octahedron edge, *i.e. c*/2. The position of **TC** and of the neighbouring **XC1** and **XC2** is thus invariant with respect to the *z* direction. Furthermore, since **XC1** and **XC2** on opposite sides of a **VOW** are bonded to Ca11 or Ca12, and these octahedra have only equatorial corners available for bonds with Si, the vertical Si_2_O_7_ groups of both **XC1** and **XC2** are at the same height (Fig. 13 ▸). Of course, it could be argued as well that this is so because of the translational symmetry along [010]. Thus, within one strip along [010] all silicate anions are fixed with respect to the *z* and of course to the *y* direction. In addition, because the relative displacements of all **XC1** and **XC2** with respect to **TC** are *c*/2, they are highly correlated and the whole strip is invariant with respect to the *y* and *z* directions. This explains why these directions are not affected by disorder and the reflections in the corresponding reciprocal directions are sharp.

**Figure 14:**
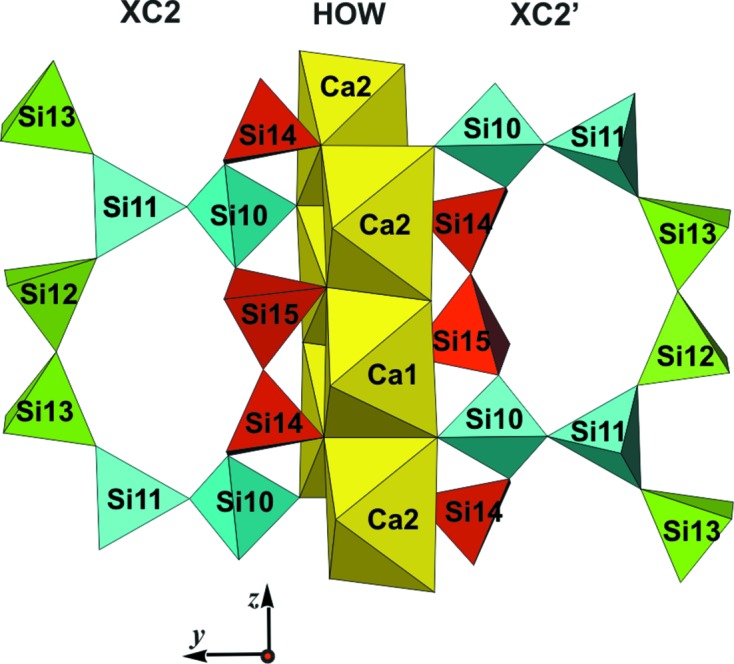
Denisovite, two **XC2** on opposite sides of an **HOW**, showing their relative displacement by *c*/4.

**Figure 15:**
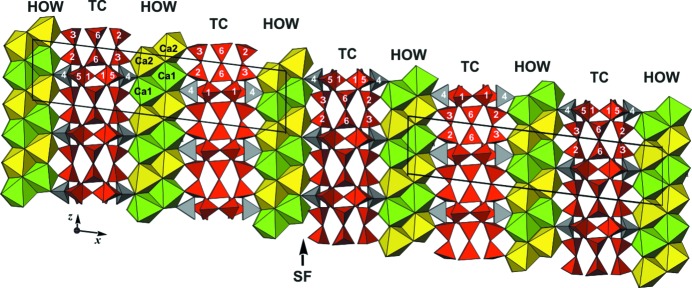
Denisovite, schematic view of a stacking fault.

**Figure 16:**
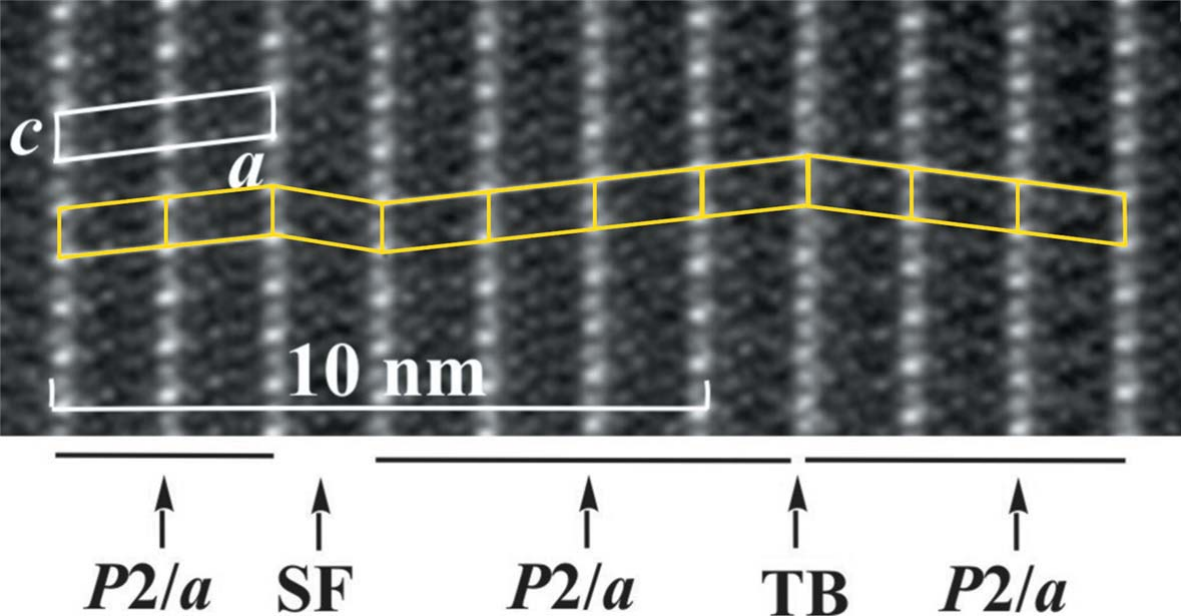
HRTEM of denisovite, showing ordered *P*12/*a*1 areas, a stacking fault (SF) and a twin boundary (TB). A unit cell is indicated in white, and the yellow rhomboids are a guide to the eyes.

The situation changes significantly when one considers two neighbouring strips in the *x* direction. The two strips are symmetrically equivalent and each one is invariant according to what has been said above. However, the silicate chains in both strips are displaced with respect to each other along the *z* direction. Two **XC2** on opposite sides of the **HOW** belong to two neighbouring strips (Fig. 14 ▸). This configuration has inversion symmetry. For the sake of simplicity, let us consider symmetrically equivalent vertical Si_2_O_7_ groups on opposite sides of the **HOW**. Each one is connected to a vertical *u*–*d* edge of one of the symmetrically equivalent octahedra. It has already been mentioned that neighbouring columns of octahedra are displaced by *c*/4 with respect to each other. Consequently, this must also be the case for the silicate chains, because they are geometrically constrained by the octahedra columns, and thus for the whole strip. However, in contrast with the octahedra columns, a given silicate chain does not have inversion symmetry and hence displacements by *c*/4 or −*c*/4 are not equivalent. In the ideal structure as determined here, all the displacements happen in one direction, say −*c*/4. In the real structure, shifts in the opposite direction will also occur with a certain probability. This could lead to twinning if coherently diffracting structures are formed on both sides of the plane where the reversal occurs. It could also lead to a new ordered structure, where shifts *c*/4 and −*c*/4 alternate regularly, or to disorder when the directions of the shifts reverse randomly, *i.e.* when the correlation between neighbouring strips is lost. Correlation can also be lost by the occurrence of stacking faults 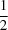
[001]. Such a stacking fault in the structure of denisovite is shown schematically in Fig. 15 ▸. Indeed, HRTEM images demonstrate that twinning and stacking faults occur already on the length scale of a few nanometres (Fig. 16 ▸). The observed strongly diffuse *l* odd diffractions (Fig. 3 ▸ *b*) are thus explained by the occurrence of reversal of *c*/4 displacements of silicate chains with respect to octahedra walls in combination with 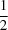
[001] stacking faults. The sharp *l* even reflections correspond to a projection of the structure into the substructure with *c*/2, which is not affected by the ambiguity in the positions of the silicate chains.

Note that, due to the geometric relationship |*a*cosβ| = *c*/2, the reversal of the monoclinic angle can be described equally well either by (100) twinning of nano-lamellae, or by keeping the orientation of the unit cell but changing the space group description to *P*12/*n*1.

### 5.4. Polytypism and the OD approach   

Denisovite presents all the characteristics typical for polytype structures, *viz.* possible decomposition into substructural units – here layers – with an ambiguity regarding their relative position, classes of diffuse and sharp reflections, diffuseness of reflections along reciprocal directions corresponding to stacking of the layers, and partial symmetries or ‘non-crystallographic’ symmetry operations. Polytypism is a widely known phenomenon for a number of Ca-bearing silicates with dreier silicate chains, for example charoite (Rozhdestvenskaya *et al.*, 2010 ▸, 2011 ▸). The interested reader is referred to the publications just mentioned and/or to comprehensive and detailed books (*e.g.* Ferraris *et al.*, 2004 ▸; Merlino, 1997 ▸). Theoretical tools for the treatment of such structures were developed by Dornberger-Schiff and her colleagues and students in the second half of the last century. An important publication discusses symmetry aspects (Dornberger-Schiff & Fichtner, 1972 ▸). It contains a list of so-called OD groupoids from which the symmetries of structures of maximum degree of order (MDO structures) can be determined.

**Figure 17:**
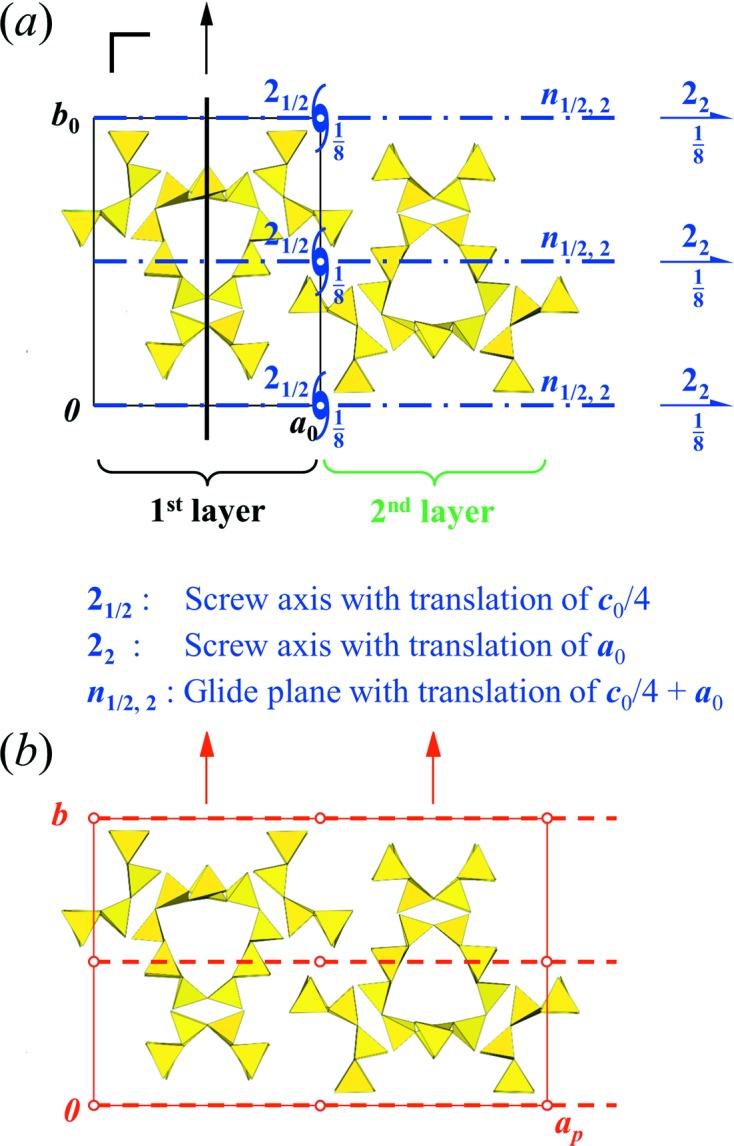
OD theory. (*a*) The symmetry elements of an isolated layer (first layer) are shown in black. The symmetry elements used to generate the second layer are shown in blue. The basis vectors of an isolated layer are *a*_0_, *b*_0_ and *c*_0_. The view is along *c*_0_. The figure displays the situation for a shift of *c*/4; in the case of a shift of −*c*/4, the *z* positions of all symmetry elements change from *z* to −*z*, the 2_1/2_ axis changes to 2_−1/2_ with a translation of −*c*_0_/4, and the *n*_1/2,2_ glide plane converts to *n*_−1/2,2_ with a translation of **a**_0_ − **c**_0_/4. (*b*) Consecutive application of the shift of −*c*/4 leads to the ordered model structure MDO1 in *P*12/*a*1, which is the main structural motif of denisovite. All depicted symmetry elements (shown in red) are crystallographic ones. All other symmetry elements from panel (*a*) are still present, but they are only local symmetries within one layer (black) or between neighbouring layers (blue). The view is along [001]. Note that the *a* axis in *P*12/*a*1 is not parallel to *a*_0_, but *a* = 2*a*_0_ − *c*_0_/2. Thereby the *n*_−1/2,2_ glide plane changes to a proper *a*-glide plane. The subscript p in *a*_p_ denotes the projection of the *a* axis.

Theoretically, the structure of denisovite can be decomposed into layers || (100), which can be considered here as consisting of the silicate anions within a strip which can be shifted with respect to the otherwise rigid framework of octahedra. The layer has basis vectors in the layer plane of *b*_0_ = *b*, *c*_0_ = *c*. The third vector, *a*_0_, represents the stacking direction. It is chosen as perpendicular to *b*_0_ and *c*_0_, and its length corresponds to the thickness of the layer, hence *a*_0_ = *a*/2 + *c*/4, with *a*_0_ = 15.44 Å and α_0_ = β_0_ = γ_0_ = 90°. An isolated undistorted layer exhibits a twofold axis along *b*_0_ and mirror planes perpendicular to *a*_0_ and *c*_0_ (Fig. 17 ▸). Hence, the layers have idealized symmetry *P*(*m*)2*m*, where the parentheses around the second position indicate the direction in which the periodicity has been lost. From the list of OD groupoid families given by Dornberger-Schiff & Fichtner (1972 ▸), a possible solution for the groupoid symbol can easily be found, which, after rearranging, reads
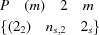
In this symbol the first line gives the layer symmetry, whereas the second line lists partial symmetry operators which transform a given layer into the next one. Their designation follows the principle of ‘normal’ space-group symbols, but appropriately adapted. Specifically, *p_q_* screw axes have translational parts *q*/*p* as in standard space groups, but *q* is no longer restricted to integer values and is not necessarily < *p*. Thus, (2_2_) in the first position of the second line indicates a twofold screw axis along [100] with a translational component 2/2, *i.e.* 1. The symbol *n*_*s*,2_ indicates a diagonal glide plane perpendicular to [010] with glide vectors *s* along the *z* direction (at this stage *s* is undetermined) and the subscript 2 indicates that the glide vector along the *x* direction is 2 × 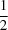 = 1, *i.e.* this glide vector transforms the first layer into the second one. The symbol 2_*s*_ in the third position of the second line indicates a screw axis || *z* with an as yet undetermined translational component *s*/2. In the specific situation of denisovite, *s* = 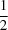 and the specified groupoid symbol becomes
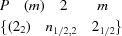
Now 2_1/2_ in the third position denotes a screw axis || [001] with a translational part of 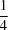, *i.e.* a shift of *c*/4. The *m* in the fourth position of the first line tells us that by applying the mirror symmetry perpendicular to *z* it does not matter whether it is 2_1/2_ or 2_−1/2_.

One of the main goals of the OD treatment is to find structures with a maximum degree of order (MDO). One of these structures, MDO1, is obtained when the sequence of the just-mentioned screw axes is …2_1/2_…2_1/2_…2_1/2_…2_1/2_… (or …2_−1/2_…2_−1/2_…2_−1/2_…2_−1/2_…). This corresponds to the situation found in our study of denisovite. Another MDO structure would be obtained by a regular reversal of shifts, *i.e.* …2_1/2_…2_−1/2_…2_1/2_…2_−1/2_… (MDO2).

Given the lattice parameters of a layer, *a*_0_, *b*_0_ and *c*_0_, and consecutive shifts …2_−1/2_…2_−1/2_… (MDO1), the resulting lattice parameters become *a* = 2*a*_0_ − *c*_0_/2, *b* = *b*_0_ and *c* = *c*_0_. From the symmetry operations *m_x_*, 2_*y*_ and *m_z_* of the individual layer (subscripts indicate the orientation), only the twofold axis 2_*y*_ parallel to *b* is maintained; the mirror planes are just partial symmetry elements (local symmetries), not crystallographic (*i.e.* global) ones. From the symmetry operations acting between the layers, given in the second line of the groupoid symbol, only the glide plane survives as a crystallographic symmetry element. In the new unit-cell setting this becomes an *a*-glide perpendicular to *b*. Additionally, there are inversion centres between the layers (Fig. 17 ▸), which are not denoted in the groupoid symbol nor in the space-group symbol [note that the layer symmetry (*m*)2*m* is non­centrosymmetric]. Correspondingly, the resulting space group of the MDO1 structure becomes *P*12/*a*1, as found experimentally.

In the case of alternating 2_1/2_ and 2_−1/2_, *i.e.* of alternating shifts of *c*/4 and −*c*/4, a similar analysis would predict an MDO2 structure with space group *P*2_1_/*m*11 and lattice parameters of *a* ≃ 30.886, *b* ≃ 19.570 and *c* ≃ 7.215 Å, β = γ = 90° and a monoclinic angle α of ∼90°. This is comparable with the situation in charoite where we have found two polytypes experimentally, *viz.* charoite-90 and charoite-96 (Rozhdestvenskaya *et al.*, 2010 ▸, 2011 ▸). Interestingly, a corresponding hypothetical ‘denisovite-90’ polytype has never been observed and many studies of charoite seem to suggest that charoite-90 is more common than charoite-96. It is not easy to see why this should be so, but one could argue that the formation of a twin boundary, *i.e.* a reversed shift of silicate chains, may also mean some degree of reorganization of the underlying octahedra walls and thus would cost some extra energy.

In denisovite, neither of the two MDO structures is experimentally observed as a pure long-range ordered polytype. All investigated crystals contained an irregular stacking, *i.e.* an irregular sequence of shifts by *c*/4 and −*c*/4. However, the stacking is not random. The probability that a shift of *c*/4 is followed by another shift of *c*/4 (or −*c*/4 followed by another −*c*/4) is considerably higher than a transition from *c*/4 to −*c*/4 or *vice versa*. Correspondingly, nano-lamellae with pure *c*/4 shifts (or pure −*c*/4 shifts) are formed, which show a local arrangement as in MDO1 over several unit cells. However, these domains, which are terminated by changes from *c*/4 to −*c*/4 or *vice versa*, are typically too small to cause visible Bragg peaks within the diffuse rods. Domains with strictly alternating shifts of *c*/4 and −*c*/4 (which would correspond to a local structure as in MDO2) have never been observed in HRTEM images. Hence the structure of denisovite can be regarded as stacking disordered, with MDO1 being representative of the preferred local structure. The description of denisovite as a periodic structure with *P*12/*a*1 symmetry gives only an idealized picture of the real structure.

### 5.5. Sites of K^+^ cations and H_2_O molecules   

The tubular chains **TC** and double chains **XC1** and **XC2** have windows of eight-membered rings, ‘8MR’ (*cf*. Fig. 6 ▸). Potassium atoms K1–K4 are located near the centres of these 8MR windows (see Fig. 12 ▸) and have nine- or tenfold co­ordination. Six O atoms of the 8MR within which the potassium atoms are located, and three O atoms of the adjacent chain, take part in the charge compensation for each of these cations, thus providing an additional link between adjacent chains.

Another potassium atom (K5) is located on the axis of the **TC** (Fig. 12 ▸). It has weaker bonds than K1–K4. The H_2_O molecule can be located within the **TC** halfway between translationally equivalent K5. This site is not fully occupied (occupancy = 0.90 O). The oxygen of the H_2_O group is linked to potassium on sites K1 and K2, located in the 8MR windows. This H_2_O group also has relatively short distances from O7 and O14, suggesting possible hydrogen bonds.

The interior of the tube formed by the **XC1** and the pair of **VOW** is almost empty (Fig. 12 ▸). Since one weak peak was found in Fourier maps, H_2_O molecules or K^+^ cations could be thought to partially occupy the position inside this tube. With regard to the calculation of the microprobe analysis of denis­ovite, which requires that the K content exceeds the 14 possible atoms in the K1–K5 sites, we infer that K^+^ is the occupant of the K6 site (Table S1 in the supporting information). The refinement of the occupancy of the K6 site gave 0.32 K. Altogether, the ratio of K:Si is 3.61:15, which is in a good agreement with the microprobe analysis of denisovite (K:Si = 3.40:15) and with the data from Rietveld refinement (3.99:15).

Finally, the refinement of the occupancies of Ca sites did not bring to light any sites for Mn or Fe atoms, so the remaining minor admixture of these atoms was inferred to occupy Ca sites statistically and to have no effect on the colour of denis­ovite, which in fact is colourless to greyish. This distinguishes denisovite from charoite, which is unique among alkaline calcium silicates in that impurity elements such as Mn, Fe and Ti always occur and are located inside one of the tubes. The various colours of charoite samples depend on these impurities. Due to its attractive colour, especially the beautiful shades of violet, and its ability to be cut and polished, charoite has become a highly appreciated decorative stone and even a semi-precious gemstone. Given its less attractive properties, denisovite is unlikely ever to become a gemstone.

### 5.6. Charge-balance mechanism and crystal chemical formula   

In order to develop the crystal chemical formula for denis­ovite, the results of the refined crystal structure and charge-balance considerations have to be taken into account. The asymmetric unit contains 44 oxygen sites (excluding the H_2_O molecules). O atoms having bonds only to two Si, or to one Si and three octahedrally coordinated cations, can be considered as completely charge compensated. O atoms bonded to one Si but to only two octahedrally coordinated cations are not completely charge-compensated. Therefore, it is reasonable to assume at these oxygen sites some degree of substitution for O^2−^ by OH^−^ groups. Two sites, O43 and O44, are bonded to four Ca octahedra but not to Si, and from bond-valence considerations these positions are likely to be occupied by OH^−^ groups. However, the multiplicity (four) of both these positions would result in eight OH^−^, whereas only four are required for charge compensation. Therefore, we conjecture that some O^2−^ replace OH^−^ at these positions and, in order to retain charge balance, some O positions in the silicate anions are replaced by OH^−^, probably those which are bonded to one Si and only two Ca/Na. The anions on the F1–F4 sites form only three bonds with cations on the Ca7, Ca8 and Ca11, Ca12 sites, and thus it is likely that both OH^−^ and F^−^ are located on these positions.

From the results of the refinement and charge-balance mechanism – and neglecting the probable replacement of some O positions by OH^−^ in the silicate anions – the structural formula of denisovite can be written as K_14.44_(Ca_41.96_Na_6.04_)_Σ=48_[(Si_6_O_17_)_6_(Si_12_O_30_)_2_]F_16_(O_0.4_OH_3.6_)·2H_2_O, *Z* = 1, or in the general case K_14+*x*_(Ca,Na,Mn,Fe)_48_[Si_60_O_162_]F_16_(O_*x*_,OH_4−*x*_)·2H_2_O. In spite of the fact that this formula differs from the average obtained by microprobe analysis, K_14.76_(Ca_38.76_Na_6.20_Mn_1.04_Fe_0.76_Al_1.08_)_Σ=47.84_Si_60.2_O_162_(F_16_O_2.06_OH_2.0_)·2H_2_O, the results are well within the limits of the minimum and maximum variations in chemical composition from the separate analyses. The calculated density based on the results of the structure refinement is 2.74 Mg m^−3^ and is thus in very good agreement with the measured density of 2.76 Mg m^−3^ (Menshikov, 1984 ▸). Finally, taking into account the results of the refinement and the conjectured widespread OH^−^/O^2−^ isomorphism, the idealized formula of denisovite is K_14_Ca_42_Na_6_[Si_60_(O,OH)_162_]F_16_(O,OH)_8_·2H_2_O.

## 6. Conclusions   

The results of the study presented here demonstrate strikingly that employing a suitably chosen combination of synergistic methods, in particular various modern methods of electron crystallography, both imaging and diffraction, and complemented by XRPD, even a complex mineral structure like that of denisovite can be determined from nano-sized crystals. The challenges posed by the studied mineral should not be underestimated. This concerned the nanometre size of the sample, its habit as very thin fibres, the inherent disorder of the nanocrystals, the still not completely mature data-collection procedure and the sheer size of the structure. The often used but rather vague term ‘complex’ has recently been given a quantification based on information theory (Krivovichev, 2013 ▸). According to this approach, denisovite with a complexity parameter of about 1990 is not only ranked among the ‘very complex’ mineral structures (complexity parameter >1000), but is among the top 1% of the most complex mineral structures known to date. Interestingly, according to this approach charoite and denisovite have almost identical complexities, despite significant differences in their structural modularity.

Irrespective of the low probability that denisovite will ever attain any importance beyond the realm of mineralogy, the mere fact that such a challenging structure could be solved makes it in some sense a landmark case, with possible impact for the study of similarly complex materials. Such materials could be expected to occur for example as a result of weathering or low-temperature alteration of natural or synthetic stone, of corrosion, or of processes occurring in the ‘critical zone’, *i.e.* the borderline where biological and geological processes interact.

## Supplementary Material

Crystal structure: contains datablock(s) denisovite_from_EDT, Denisovit_from_XRPD. DOI: 10.1107/S2052252517002585/fc5016sup1.cif

Structure factors: contains datablock(s) denisovite_from_EDT. DOI: 10.1107/S2052252517002585/fc5016denisovite_from_EDTsup2.hkl

Rietveld powder data: contains datablock(s) Denisovit_from_XRPD. DOI: 10.1107/S2052252517002585/fc5016Denisovit_from_XRPDsup3.rtv

Additional tables and figures. DOI: 10.1107/S2052252517002585/fc5016sup4.pdf

CCDC references: 1532655, 1532656

## Figures and Tables

**Table 1:** Crystallographic data, and experimental and refinement parameters for the denisovite structure determined by electron diffraction (ED) and X-ray powder diffraction (XRPD)

	ED	XRPD
*a* (Å)	31.024 (1)	31.0964 (8)
*b* (Å)	19.554 (1)	19.5701 (5)
*c* (Å)	7.1441 (5)	7.21526 (12)
β (°)	95.99 (3)	96.6669 (6)
*V* (Å^3^)	4310.3 (9)	4361.23 (18)
Space group	*P*12/*a*1	*P*12/*a*1
*D*_calc_ (Mg m^−3^)	2.74	2.71
*F*_000_	3341	3341
Crystal size (nm)	1000 × 200 × 200	
λ (Å)	0.0251	1.5406
2θ_max_ (°)	1.22	100
(sinθ/λ)_max_	0.423	0.497
(*h*, *k*, *l*)_max_	25, 15, 5	30, 19, 7
Total reflections	6809	
Resolution (Å)	1.20	
*R*_σ_	0.266	
Completeness (%)	97	
*R*_eq_	0.179	
Unique with |*F*_o_| > 4.0σ_*F*_	2454	
No. of atom sites	82	82
No. of free parameters	323	288
*R*_1_^ED^ †	0.336	
*R*_wp_		0.0383
*R*_p_		0.0296
*R*_wp_′‡		0.158
*R*_p_′‡		0.174
GOF§	8.7	2.17

† *R*_1_^ED^ = Σ||*F*_o_| − |*F*_c_||/Σ|*F*_o_|.

‡ The values *R*_p_′ and *R*_wp_′ are background-subtracted.

§ GOF (goodness of fit) = {Σ[*w*(*F*_o_^2^ − *F*_c_^2^)]/(*n* − *p*)}^1/2^, where *n* is the number of reflections and *p* is the number of refined parameters.

**Table 2:** Selected bond lengths (Å) in the denisovite structure

Ca1—O5	2.26 (4)	Ca2—O6	2.24 (4)	Ca3—O41	2.23 (4)	Ca4—O20	2.19 (4)
Ca1—O27	2.27 (4)	Ca2—O38	2.25 (4)	Ca3—O22	2.24 (4)	Ca4—O21	2.35 (3)
Ca1—O10	2.29 (4)	Ca2—O27	2.35 (4)	Ca3—O2	2.27 (4)	Ca4—O5	2.36 (3)
Ca1—O38	2.36 (4)	Ca2—O39	2.37 (4)	Ca3—O6	2.48 (4)	Ca4—O40	2.40 (4)
Ca1—O39	2.45 (4)	Ca2—O39	2.39 (4)	Ca3—O40	2.48 (4)	Ca4—O41	2.44 (4)
Ca1—O38	2.60 (4)	Ca2—O10	2.46 (4)	Ca3—O20	2.53 (4)	Ca4—O2	2.62 (4)
Average	2.37		2.34		2.37		2.39
							
Ca5—F2	2.19 (4)	Ca6—F1	2.17 (4)	Ca7—O43	2.18 (5)	Ca8—O44	2.25 (4)
Ca5—O40	2.33 (3)	Ca6—O22	2.32 (4)	Ca7—O32	2.28 (5)	Ca8—F2	2.25 (5)
Ca5—O44	2.38 (3)	Ca6—O41	2.33 (4)	Ca7—O32	2.32 (5)	Ca8—O33	2.30 (4)
Ca5—O21	2.39 (4)	Ca6—O21	2.36 (4)	Ca7—O33	2.37 (6)	Ca8—O33	2.31 (4)
Ca5—O22	2.40 (4)	Ca6—O43	2.42 (3)	Ca7—F1	2.44 (5)	Ca8—O32	2.34 (4)
Ca5—F1	2.45 (4)	Ca6—F2	2.50 (4)	Ca7—O44	2.59 (5)	Ca8—O43	2.59 (4)
Average	2.36		2.35		2.36		2.34
							
Ca9—O15	2.19 (4)	Ca10—F4	2.30 (3)	Ca11—O23	2.18 (4)	Ca12—O24	2.21 (4)
Ca9—O16	2.26 (3)	Ca10—O15	2.31 (4)	Ca11—O24	2.32 (4)	Ca12—F3	2.25 (4)
Ca9—F3	2.32 (3)	Ca10—F3	2.34 (3)	Ca11—O44	2.37 (4)	Ca12—O23	2.33 (4)
Ca9—F4	2.33 (3)	Ca10—O28	2.36 (4)	Ca11—O35	2.39 (5)	Ca12—O34	2.36 (4)
Ca9—O34	2.45 (4)	Ca10—O16	2.39 (3)	Ca11—O34	2.42 (4)	Ca12—O43	2.39 (4)
Ca9—O28	2.53 (4)	Ca10—O35	2.44 (4)	Ca11—F4	2.49 (4)	Ca12—O35	2.42 (4)
Average	2.35		2.36		2.36		2.33
							
Si1—O4	1.53 (5)	Si2—O5	1.58 (5)	Si3—O4	1.57 (5)	Si4—O8	1.55 (5)
Si1—O1	1.59 (5)	Si2—O7	1.60 (5)	Si3—O6	1.58 (5)	Si4—O10	1.56 (6)
Si1—O2	1.65 (5)	Si2—O8	1.61 (5)	Si3—O9	1.63 (4)	Si4—O11	1.70 (6)
Si1—O3	1.69 (5)	Si2—O3	1.67 (5)	Si3—O7	1.66 (4)	Si4—O9	1.71 (6)
Average	1.62		1.61		1.61		1.63
							
Si5—O12	1.55 (4)	Si6—O12	1.59 (5)	Si7—O18	1.59 (5)	Si8—O21	1.58 (6)
Si5—O11	1.64 (5)	Si6—O15	1.62 (6)	Si7—O17	1.61 (4)	Si8—O18	1.58 (6)
Si5—O16	1.67 (4)	Si6—O13	1.63 (5)	Si7—O20	1.62 (4)	Si8—O25	1.66 (6)
Si5—O13	1.67 (4)	Si6—O14	1.64 (4)	Si7—O19	1.65 (5)	Si8—O23	1.67 (5)
Average	1.63		1.62		1.62		1.62
							
Si9—O24	1.54 (4)	Si10—O26	1.57 (5)	Si11—O30	1.58 (5)	Si12—O34	1.55 (5)
Si9—O22	1.56 (4)	Si10—O36	1.58 (4)	Si11—O26	1.60 (5)	Si12—O32	1.58 (5)
Si9—O25	1.61 (5)	Si10—O27	1.63 (4)	Si11—O28	1.63 (5)	Si12—O31	1.64 (4)
Si9—O19	1.73 (4)	Si10—O37	1.70 (5)	Si11—O29	1.64 (5)	Si12—O30	1.70 (5)
Average	1.61		1.62		1.61		1.62
							
Si13—O35	1.56 (4)	Si14—O41	1.56 (4)	Si15—O36	1.56 (5)	*W*1—O14	2.80 (5)
Si13—O33	1.56 (4)	Si14—O42	1.60 (5)	Si15—O38	1.60 (5)	*W*1—O7 ×2	2.86 (4)
Si13—O29	1.64 (4)	Si14—O37	1.61 (4)	Si15—O42	1.64 (5)	*W*1—K1	2.96 (6)
Si13—O31	1.71 (4)	Si14—O39	1.65 (4)	Si15—O40	1.69 (5)	*W*1—K2 ×2	3.20 (4)
Average	1.62		1.61		1.62		
							
K1—O3 ×2	2.87 (4)	K2—O13	2.75 (5)	K3—O11	2.69 (6)	K5—O13 ×2	3.16 (5)
K1—O4 ×2	2.89 (3)	K2—O26	2.75 (5)	K3—F1	2.73 (5)	K5—O9 ×2	3.23 (3)
K1—O17	2.89 (6)	K2—O9	2.89 (4)	K3—O31	2.86 (5)	K5—O8 ×2	3.30 (3)
K1—*W*1	2.96 (3)	K2—O8	2.90 (4)	K3—O36	2.91 (5)	K5—O3 ×2	3.43 (5)
K1—O7 ×2	3.01 (5)	K2—O14	2.92 (4)	K3—O30	2.94 (6)	K5—O4 ×2	3.52 (5)
K1—O20 ×2	3.18 (4)	K2—O28	2.93 (5)	K3—O37	3.06 (6)	K5—*W*1 ×2	3.57 (5)
Average	2.97	K2—O7	3.04 (5)	K3—O42	3.07 (5)	K5—O12	3.60 (6)
		K2—O12	3.16 (5)	K3—O29	3.09 (5)	Average	3.40
		K2—*W*1	3.20 (5)	K3—O10	3.27 (5)		
		Average	2.95	Average	2.96		
K4—O1	2.70 (9)					K6—F4 ×2	2.43 (5)
K4—O25 ×2	2.70 (4)					K6—O23 ×2	3.38 (3)
K4—O19 ×2	2.80 (3)					K6—O25 ×2	3.52 (5)
K4—O18 ×2	2.96 (3)					K6—O24 ×2	3.69 (4)
K4—O2 ×2	3.09 (4)					Average	3.25
Average	2.87						
