# Supplementary material for: The structure of denisovite, a fibrous nanocrystalline polytypic disordered ‘very complex’ silicate, studied by a synergistic multi-disciplinary approach employing methods of electron crystallography and X-ray powder diffraction
Source: IUCrJ. 2017 Mar 8;4(Pt 3):223–42. doi: 10.1107/S2052252517002585 (PMC5414397; doi:10.1107/S2052252517002585)
Supplement: Supplementary file 4 [file m-04-00223-sup4.pdf]

IUCrJ

Volume 4 (2017)

Supporting information for article:

The structure of denisovite, a fibrous nano-crystalline polytypic disordered `very complex' silicate, studied by a synergistic multi-disciplinary approach employing methods of electron crystallography and X-ray powder diffraction

Ira V. Rozhdestvenskaya, Enrico Mugnaioli, Marco Schowalter, Martin U. Schmidt, Michael Czank, Wulf Depmeier and Andreas Rosenauer

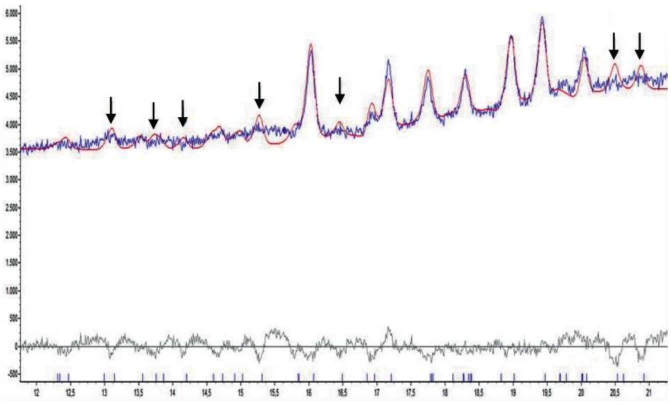

Figure S1. Exemplary broad peaks in the XRPD region 13° < 2θ < 22°. Blue line: experimental data; red line: Rietveld refined model. Residual is shown at the bottom. The most obvious broad peaks are indicated by a black arrow: 2 theta = 13.1° (1-1-1), 13.7° (111 and 2-1-1), 14.2° (201), 15.4° (021 and 1-2-1), 16.5° (4-1-1 and 311), 20.5° (511), 20.9° (331 and 4-3-1).

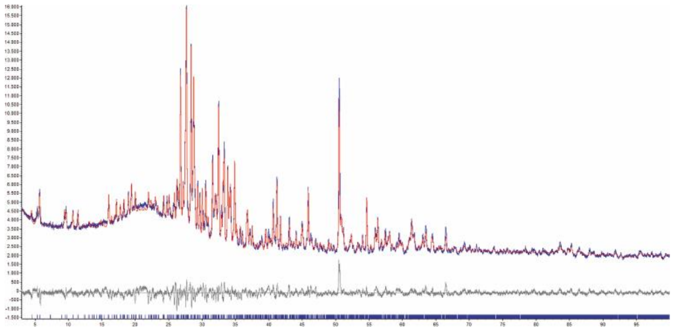

Figure S2. Rietveld plot for the Rietveld refinement carried out without any correction for preferred orientation.

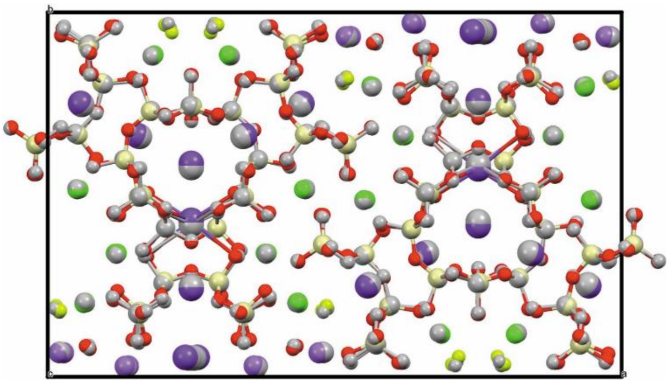

Figure S3. Comparison of the crystal structures determined from powder data by Rietveld refinement with correction for preferred orientation (coloured) and without correction for preferred orientation (grey). Colour codes: Si beige, O red, Ca and Na green, K purple, F yellow.

Table S1 Atom coordinates, isotropic displacement parameters (Å^2) and site occupancies of denisovite structure on the basis of EDT data.

Site	Atom	N[a]	*x/a*	*y/b*	*z/c*	$B_{iso/eqv}$[b]	Occupancy[c]
K(1)	K	2	0.25	0.427(3)	0.5	7.1(15)	
K(2)	K	4	0.162(1)	0.666(2)	0.452(5)	3.5(12)	0.90(7)
K(3)	K	4	0.055(2)	0.741(3)	0.907(6)	7.7(17)	0.89(8)
K(4)	K	2	0.25	0.235(2)	0	4.1(11)	
K(5)	K	2	0.25	0.583(3)	0	5.3(12)	
K(6)	K	4	0.250(5)	0.046(10)	0.253(9)	11.4(76)	0.32(7)
Ca(1)	Ca	4	0.051(1)	0.515(2)	0.150(4)	5.0(8)	
Ca(2)	Ca	4	0.0492(9)	0.518(2)	0.650(4)	3.2(7)	
Ca(3)	Ca	4	0.1204(9)	0.332(2)	0.689(4)	4.0(8)	
Ca(4)	Ca	4	0.1180(8)	0.332(2)	0.189(4)	2.7(7)	
Ca(5)	Ca	4	0.0620(8)	0.203(2)	0.901(4)	2.8(7)	
Ca(6)	Ca	4	0.0664(8)	0.204(2)	0.415(4)	2.4(6)	
Ca(7)	Ca	4	0.017(2)	0.064(2)	0.129(6)	5.7(17)	0.44(5)
Na(1)	Na	4	0.017(2)	0.064(2)	0.129(6)	5.7(17)	0.56(5)
Ca(8)	Ca	4	0.0174(9)	0.070(2)	0.634(4)	3.4(11)	0.67(5)
Na(2)	Na	4	0.0174(9)	0.070(2)	0.634(4)	3.4(11)	0.33(5)
Ca(9)	Ca	4	0.1841(7)	0.878(1)	0.213(3)	1.3(6)	
Ca(10)	Ca	4	0.1818(8)	0.879(1)	0.713(3)	1.8(6)	
Ca(11)	Ca	4	0.1376(9)	0.027(2)	0.946(4)	2.3(10)	0.78(6)
Na(3)	Na	4	0.1376(9)	0.027(2)	0.946(4)	2.3(10)	0.22(6)
Ca(12)	Ca	4	0.1351(8)	0.026(2)	0.442(4)	2.5(10)	0.60(5)
Na(4)	Na	4	0.1351(8)	0.026(2)	0.442(4)	2.5(10)	0.40(5)
Si(1)	Si	4	0.203(1)	0.405(2)	0.953(6)	3.7(10)	
Si(2)	Si	4	0.156(2)	0.493(2)	0.235(6)	4.8(11)	

Si(3)	Si	4	0.155(1)	0.496(2)	0.669(5)	3.0(10)
Si(4)	Si	4	0.131(2)	0.597(3)	0.955(7)	6.9(14)
Si(5)	Si	4	0.1790(9)	0.730(2)	0.961(4)	1.5(7)
Si(6)	Si	4	0.248(2)	0.752(2)	0.712(6)	4.2(10)
Si(7)	Si	4	0.199(1)	0.262(2)	0.483(5)	3.7(10)
Si(8)	Si	4	0.157(2)	0.173(3)	0.179(7)	8.1(15)
Si(9)	Si	4	0.1541(9)	0.169(2)	0.740(4)	1.4(7)
Si(10)	Si	4	0.048(1)	0.661(2)	0.372(5)	3.7(10)
Si(11)	Si	4	0.096(2)	0.801(2)	0.413(6)	5.0(11)
Si(12)	Si	4	0.075(1)	0.913(2)	0.124(5)	3.6(10)
Si(13)	Si	4	0.0722(9)	0.914(2)	0.684(4)	2.7(7)
Si(14)	Si	4	0.027(1)	0.360(2)	0.385(5)	2.2(9)
Si(15)	Si	4	0.022(1)	0.372(2)	0.935(5)	3.9(10)
O(1)	O	2	0.25	0.372(4)	0	8.8(73)
O(2)	O	4	0.173(1)	0.335(2)	0.934(5)	8.3(23)
O(3)	O	4	0.195(1)	0.446(2)	0.155(5)	5.1(17)
O(4)	O	4	0.189(1)	0.447(2)	0.777(4)	9.1(55)
O(5)	O	4	0.1121(8)	0.4529(9)	0.219(3)	4.3(15)
O(6)	O	4	0.111(1)	0.458(1)	0.679(4)	1.1(10)
O(7)	O	4	0.167(1)	0.510(2)	0.453(3)	7.8(22)
O(8)	O	4	0.153(1)	0.569(1)	0.144(3)	2.0(11)
O(9)	O	4	0.156(1)	0.573(1)	0.761(4)	5.6(17)
O(10)	O	4	0.081(1)	0.579(1)	0.930(5)	9.3(24)
O(11)	O	4	0.134(1)	0.684(2)	0.963(7)	9.2(56)
O(12)	O	4	0.209(1)	0.745(2)	0.144(4)	9.2(33)
O(13)	O	4	0.207(1)	0.710(2)	0.781(5)	8.0(22)

O(14)	O	2	0.25	0.721(2)	0.5	8.4(54)
O(15)	O	4	0.249(1)	0.834(2)	0.280(5)	1.7(10)
O(16)	O	4	0.160(1)	0.810(1)	0.965(3)	4.5(16)
O(17)	O	2	0.25	0.280(1)	0.5	5.5(24)
O(18)	O	4	0.193(1)	0.213(1)	0.305(5)	3.9(15)
O(19)	O	4	0.199(1)	0.208(2)	0.661(4)	5.3(17)
O(20)	O	4	0.169(1)	0.328(1)	0.426(4)	9.3(29)
O(21)	O	4	0.112(1)	0.213(1)	0.174(5)	1.7(12)
O(22)	O	4	0.115(1)	0.218(1)	0.683(4)	1.8(12)
O(23)	O	4	0.159(1)	0.088(1)	0.194(4)	8.9(23)
O(24)	O	4	0.152(1)	0.093(1)	0.690(4)	9.0(29)
O(25)	O	4	0.171(1)	0.175(1)	0.960(5)	4.2(16)
O(26)	O	4	0.081(1)	0.722(1)	0.400(4)	4.7(16)
O(27)	O	4	0.068(1)	0.585(1)	0.400(5)	4.0(15)
O(28)	O	4	0.148(1)	0.814(2)	0.460(5)	9.3(24)
O(29)	O	4	0.074(1)	0.838(1)	0.583(4)	6.5(19)
O(30)	O	4	0.074(1)	0.835(2)	0.228(5)	5.4(17)
O(31)	O	4	0.066(1)	0.887(1)	0.907(3)	5.4(17)
O(32)	O	4	0.0294(9)	0.947(1)	0.134(4)	5.1(17)
O(33)	O	4	0.028(1)	0.953(1)	0.631(5)	4.2(15)
O(34)	O	4	0.119(1)	0.949(1)	0.189(5)	6.0(18)
O(35)	O	4	0.116(1)	0.950(2)	0.692(5)	6.1(18)
O(36)	O	4	0.015(1)	0.663(1)	0.188(3)	9.3(45)
O(37)	O	4	0.015(1)	0.675(1)	0.545(5)	7.4(22)
O(38)	O	4	0.029(1)	0.450(2)	0.879(4)	2.9(13)
O(39)	O	4	0.024(1)	0.444(1)	0.395(5)	1.3(8)

O(40)	O	4	0.066(1)	0.322(1)	0.917(4)	6.2(18)	
O(41)	O	4	0.071(1)	0.323(1)	0.440(5)	2.4(13)	
O(42)	O	4	0.021(1)	0.357(1)	0.160(5)	3.9(15)	
O(43)	O	4	0.067(1)	0.081(1)	0.365(4)	9.4(26)	
O(44)	O	4	0.070(1)	0.082(1)	0.874(5)	3.4(14)	
F(1)	F	4	0.020(1)	0.188(1)	0.171(4)	9.4(25)	
F(2)	F	4	0.015(1)	0.185(2)	0.657(4)	6.8(20)	
F(3)	F	4	0.192(1)	0.953(1)	0.465(4)	7.6(20)	
F(4)	F	4	0.201(1)	0.949(1)	0.967(4)	8.0(22)	
W(1)	O	2	0.25	0.578(2)	0.5	7.0(55)	0.90(2)

a) N: multiplicity. *b*) $U_{eq} = 1/3[B_{11}\, a^{*2}\, a^2 + \ldots + 2B_{23}\, b^*\, c^*\, b\, c\, \cos(\alpha)]$. *c*) Occupancy of mixed sites only is shown. All other sites are fully occupied by the respective element.

Table S2 Bond Valence Sums (BVS) in valence units (v.u.) calculated according to http://www.iucr.org/__data/assets/file/0007/126574/bvparm2016.cif. Water was neglected. BVS Si1 – Si15: mean: 4.11(8), max: 4.23, min: 3.94; BVS Ca1 – Ca12: mean: 2.07(10), max: 2.23, min: 1.90; BVS K1 – K6: mean: 0.95 (31), max: 1.36, min: 0.42 (K6 position is characterized by disordered); BVS O1 – O42: mean: 2.11(26), max: 2.75, min: 1.65; BVS O43 – O44: mean: 1.35(2), max: 1.36, min: 1.33; BVS F1 – F4: mean: 1.04 (28), max: 1.45, min: 0.86.

	Si1	Si2	Si3	Si4	Si5	Si6	Si7	Si8	Si9	Si10	Si11	Si12	Si13	Si14	Si15
O1	2.20[a]														
O2	0.93														
O3	0.84	0.88													
O4	1.29		1.16												
O5		1.13													
O6			1.13												
O7		1.07	0.91												
O8		1.04		1.22											
O9			0.98	0.79											
O10				1.19											
O11				0.81	0.96										
O12					1.22	1.10									
O13					0.88	0.98									
O14						1.92[a]									
O15						1.01									
O16					0.88										
O17							2.08[a]								
O18							1.10	1.13							
O19							0.93		0.75						
O20							1.01								
O21								1.13							
O22									1.19						
O23								0.88							
O24									1.25						
O25								0.91	1.04						
O26										1.16	1.07				
O27										0.98					
O28											0.98				
O29											0.96		0.96		
O30											1.13	0.81			
O31												0.96	0.79		
O32												1.13			
O33													1.19		
O34												1.22			
O35													1.19		

O36										1.13					1.19
O37										0.81				1.04	
O38															1.07
O39														0.93	
O40															0.84
O41														1.19	
O42														1.07	0.96
O43															
O44															
F1															
F2															
F3															
F4															
Σ	4.16	4.12	4.18	4.01	3.94	4.05	4.08	4.05	4.23	4.08	4.14	4.12	4.13	4.23	4.06

	Ca1	Ca2	Ca3	Ca4	Ca5	Ca6	Ca7	Ca8	Ca9	Ca10	Ca11	Ca12
O1												
O2			0.44	0.17								
O3												
O4												
O5	0.45			0.35								
O6		0.48	0.25									
O7												
O8												
O9												
O10	0.42	0.26										
O11												
O12												
O13												
O14												
O15									0.55	0.40		
O16									0.45	0.32		
O17												
O18												
O19												
O20			0.22	0.55								
O21				0.36	0.32	0.35						
O22			0.48		0.31	0.39						
O23											0.56	0.37
O24											0.39	0.52
O25												
O26												

O27	0.44	0.36										
O28									0.22	0.35		
O29										0.35		
O30												
O31												
O32							0.43<br>0.39	0.36				
O33							0.34	0.41<br>0.40				
O34									0.27		0.29	0.35
O35										0.28	0.32	0.29
O36												
O37												
O38	0.35<br>0.18	0.47										
O39	0.27	0.34<br>0.32										
O40			0.25	0.31	0.37							
O41			0.49	0.28		0.37						
O42												
O43						0.29	0.56	0.19				0.32
O44					0.33		0.19	0.47			0.34	
F1					0.19	0.41	0.20					
F2					0.39	0.17		0.33				
F3									0.27	0.26		0.33
F4									0.27	0.29	0.17	
Σ	2.11	2.23	2.13	2.02	1.91	1.98	2.11	2.16	2.03	1.90	2.07	2.18

	K1	K2	K3	K4	K5	K6		Σ
O1				0.22				2.42
O2				0.08<br>0.08				1.70
O3	0.14<br>0.14				0.03<br>0.03			2.06
O4	0.13<br>0.13				0.02<br>0.02			2.75
O5								1.93
O6								1.86
O7	0.09<br>0.09	0.09						2.25
O8		0.13			0.04			2.47

					0.04			
O9		0.13			0.05<br>0.05			2.00
O10			0.05					1.92
O11			0.22					1.99
O12		0.09			0.02			2.43
O13		0.19			0.06<br>0.06			2.17
O14		0.12						2.04
O15								1.96
O16								1.65
O17	0.13							2.21
O18				0.11<br>0.11				2.45
O19				0.16<br>0.16				2.00
O20	0.06<br>0.06							1.90
O21								2.16
O22								2.37
O23						0.03<br>0.03		1.87
O24						0.01<br>0.01		2.18
O25				0.22<br>0.22		0.02<br>0.02		2.43
O26		0.19						2.42
O27								1.78
O28		0.12						1.67
O29			0.08					2.35
O30			0.11					2.05
O31			0.14					1.89
O32								2.31
O33								2.34
O34								2.13
O35								2.08
O36			0.12					2.44
O37			0.08					1.93
O38								2.07
O39								1.86
O40								1.77
O41								2.33
O42			0.08					2.11

O43								1.36
O44								1.33
F1			0.14					0.94
F2								0.89
F3								0.86
F4						0.36<br>0.36		1.45
Σ	0.97	1.06	1.02	1.36	0.42	0.84		

a) O1, O14 and O17 make bonds with two symmetrically equivalent Si.

Table S3 Atom coordinates, isotropic displacement parameters (Å^2) and site occupancies of denisovite structure on the basis of XRPD Rietveld refinement.

Site	Atom	N[a]	*x/a*	*y/b*	*z/c*	$B_{iso/eqv}$[b]	Occupancy[c]
K1	K	2	0.25	0.4295(12)	0.5	6	0.775(17)
K2	K	4	0.15708(47)	0.66153(60)	0.45455(95)	6	1.000(13)
K3	K	4	0.05723(48)	0.75018(68)	0.90605(99)	6	1.000(12)
K4	K	2	0.25	0.24265(87)	0	6	0.935(17)
K5	K	2	0.25	0.5857(14)	0	6	0.677(18)
K6	K	4	0.26042(59)	0.05335(78)	0.2904(20)	6	0.799(12)
Ca1	Ca	4	0.05474(41)	0.51434(49)	0.1374(10)	3	
Ca2	Ca	4	0.05033(45)	0.51391(51)	0.65198(99)	3	
Ca3	Ca	4	0.12154(43)	0.33951(51)	0.6683(11)	3	
Ca4	Ca	4	0.11724(39)	0.33942(53)	0.2034(11)	3	
Ca5	Ca	4	0.06269(40)	0.20077(50)	0.89550(96)	3	
Ca6	Ca	4	0.06524(43)	0.20536(50)	0.41229(97)	3	
Ca7	Ca	4	0.02746(49)	0.06611(65)	0.1222(12)	3	0.44
Na1	Na	4	0.02746(49)	0.06611(65)	0.1222(12)	3	0.56
Ca8	Ca	4	0.01970(47)	0.06654(58)	0.6338(12)	3	0.67
Na2	Na	4	0.01970(47)	0.06654(58)	0.6338(12)	3	0.33
Ca9	Ca	4	0.18552(42)	0.88377(51)	0.20437(92)	3	
Ca10	Ca	4	0.18296(44)	0.87960(54)	0.72061(91)	3	
Ca11	Ca	4	0.13482(43)	0.02877(59)	0.9646(11)	3	0.78
Na3	Na	4	0.13482(43)	0.02877(59)	0.9646(11)	3	0.22
Ca12	Ca	4	0.14048(50)	0.02979(60)	0.4314(12)	3	0.6
Na4	Na	4	0.14048(50)	0.02979(60)	0.4314(12)	3	0.4
Si1	Si	4	0.20542(28)	0.40833(40)	0.96642(81)	3	

Si2	Si	4	0.15780(27)	0.49383(42)	0.23054(96)	3
Si3	Si	4	0.16510(27)	0.49647(39)	0.6669(10)	3
Si4	Si	4	0.13375(26)	0.59496(42)	0.93322(83)	3
Si5	Si	4	0.17911(27)	0.72863(40)	0.97002(83)	3
Si6	Si	4	0.25153(26)	0.74094(39)	0.7177(11)	3
Si7	Si	4	0.20268(28)	0.26109(40)	0.46651(82)	3
Si8	Si	4	0.15998(27)	0.17874(38)	0.1794(10)	3
Si9	Si	4	0.16047(28)	0.17296(42)	0.71683(94)	3
Si10	Si	4	0.05258(28)	0.66414(42)	0.39424(84)	3
Si11	Si	4	0.09456(26)	0.80639(43)	0.42207(84)	3
Si12	Si	4	0.07251(27)	0.91231(39)	0.13631(94)	3
Si13	Si	4	0.07017(26)	0.90571(40)	0.69046(94)	3
Si14	Si	4	-0.02091(27)	0.63758(40)	0.64290(96)	3
Si15	Si	4	-0.01917(26)	0.63839(40)	0.07847(99)	3
O1	O	2	0.25	0.36977(96)	1	3
O2	O	4	0.16868(48)	0.34847(78)	0.9283(21)	3
O3	O	4	0.20302(40)	0.45958(62)	0.1647(14)	3
O4	O	4	0.20345(43)	0.45707(63)	0.7825(15)	3
O5	O	4	0.11433(46)	0.45010(82)	0.1876(21)	3
O6	O	4	0.12426(46)	0.44385(86)	0.6604(23)	3
O7	O	4	0.17415(60)	0.50431(86)	0.4494(12)	3
O8	O	4	0.15036(54)	0.56401(56)	0.1372(16)	3
O9	O	4	0.15771(49)	0.57130(58)	0.7500(15)	3
O10	O	4	0.08109(41)	0.5834(11)	0.8977(20)	3
O11	O	4	0.14036(44)	0.67698(52)	0.9407(18)	3
O12	O	4	0.20359(39)	0.71942(77)	1.1863(13)	3

O13	O	4	0.20937(35)	0.70885(73)	0.8043(15)	3
O14	O	2	0.25	0.7063(12)	0.5	3
O15	O	4	0.25065(72)	0.82790(62)	0.7280(27)	3
O16	O	4	0.16249(67)	0.81386(63)	0.9450(20)	3
O17	O	2	0.25	0.2897(10)	0.5	3
O18	O	4	0.20220(41)	0.20877(63)	0.2814(16)	3
O19	O	4	0.19785(42)	0.22235(62)	0.6714(15)	3
O20	O	4	0.17322(51)	0.33010(71)	0.4150(22)	3
O21	O	4	0.12104(47)	0.23503(79)	0.1636(25)	3
O22	O	4	0.11440(45)	0.21067(95)	0.6511(21)	3
O23	O	4	0.14803(57)	0.10173(65)	0.2499(22)	3
O24	O	4	0.16900(64)	0.10360(58)	0.5971(22)	3
O25	O	4	0.16437(66)	0.17600(82)	0.9492(11)	3
O26	O	4	0.08520(47)	0.72796(51)	0.3947(18)	3
O27	O	4	0.08454(61)	0.59780(71)	0.4179(19)	3
O28	O	4	0.14861(41)	0.8201(11)	0.4540(20)	3
O29	O	4	0.07684(50)	0.83025(58)	0.6193(14)	3
O30	O	4	0.06690(52)	0.84128(57)	0.2460(16)	3
O31	O	4	0.07365(58)	0.88866(87)	0.9173(12)	3
O32	O	4	0.02600(47)	0.95543(88)	0.1367(20)	3
O33	O	4	0.02332(47)	0.93690(89)	0.6047(23)	3
O34	O	4	0.11637(50)	0.96139(85)	0.2035(21)	3
O35	O	4	0.11088(50)	0.96071(82)	0.6743(20)	3
O36	O	4	0.02260(39)	0.67205(82)	0.1830(15)	3
O37	O	4	0.02190(35)	0.66869(71)	0.5596(16)	3
O38	O	4	-0.02444(71)	0.55578(63)	0.1125(22)	3

O39	O	4	-0.01962(74)	0.55618(63)	0.5900(23)	3	
O40	O	4	-0.06330(47)	0.68432(87)	0.1201(21)	3	
O41	O	4	-0.06378(46)	0.68218(93)	0.5759(21)	3	
O42	O	4	-0.01125(59)	0.65233(93)	-0.1336(12)	3	
O43	O	4	0.07276(80)	0.0765(12)	0.4090(22)	3	
O44	O	4	0.06643(81)	0.0842(13)	0.9202(22)	3	
F1	F	4	0.01438(90)	0.1944(10)	0.1495(21)	3	
F2	F	4	0.02203(96)	0.1818(12)	0.6231(22)	3	
F3	F	4	0.20422(85)	0.9658(11)	0.4949(20)	3	
F4	F	4	0.21619(83)	0.9449(11)	0.9670(20)	3	
O1w	O	2	0.25	0.5655(22)	0.5	3	0.9

a) N: multiplicity. *b*) $U_{eq} = 1/3[B_{11}\, a^{*2}\, a^2 + \ldots + 2B_{23}\, b^*\, c^*\, b\, c\, \cos(\alpha)]$. *c*) Occupancy of mixed sites only is shown. All other sites are fully occupied by the respective element.

Table S4 Crystallographic data from Rietveld refinements carried out with and without a correction for preferred orientation (P.O.).

	Final data (with correction for P.O.)	Rietveld refinement without correction for P.O.
a (Å)	31.0964(8)	31.0969(9)
b (Å)	19.5701(5)	19.5757(6)
c (Å)	7.21526(12)	7.21567(15)
β (°)	96.6669(6)	96.638(6)
V (Å^3)	4361.23(18)	4363.05(21)
Space group	*P* 1 2/*a* 1	*P* 1 2/*a* 1
D_{calc} (g/cm^3)	2.71	2.71
F_{000}	3341	3341
λ (Å)	1.5406	1.5406
$2\theta_{max}$ (°)	100	100
$(\sin\theta/\lambda)_{max}$	0.497	0.497
$(h,k,l)_{max}$	30, 19, 7	30, 19, 7
N atom sites	82	82
N free parameters	288	288
R_p	0.0296	0.0333
R_{wp}' [a]	0.158	0.176
R_p' [a]	0.174	0.189
GOF [b]	2.17	2.50

a) The values R_p' and R_{wp}' are background subtracted. b) GOF = $\{\Sigma[w(F_o^2 - F_c^2)]/(n - p)\}^{1/2}$, where *n* is the number of reflections and *p* is the number of refined parameters.
